# PKCε phosphorylates MIIP and promotes colorectal cancer metastasis through inhibition of RelA deacetylation

**DOI:** 10.1038/s41467-017-01024-2

**Published:** 2017-10-16

**Authors:** Tao Chen, Jingjie Li, Meidong Xu, Qin Zhao, Yingyong Hou, Liqing Yao, Yunshi Zhong, Ping-Chieh Chou, Wei Zhang, Pinghong Zhou, Yuhui Jiang

**Affiliations:** 1 Endoscopy Center and Endoscopy Research Institute, Zhongshan Hospital, Fudan University, Shanghai, 200032 China; 2 The Institute of Cell Metabolism, Shanghai Key Laboratory of Pancreatic disease, Shanghai General Hospital, School of Medicine, Shanghai Jiaotong University, Shanghai, 201620 China; 3 Department of Pathology, Zhongshan Hospital, Fudan University, Shanghai, 200032 China; 4 Department of Cancer Biology, Comprehensive Cancer Center of Wake Forest Baptist Medical Center, Winston-Salem, NC 27157 USA

## Abstract

EGFR signaling is implicated in NF-κB activation. However, the concrete mechanisms by which the core transducer of NF-κB signaling pathway, RelA/p65 is regulated under EGFR activation remains to be further clarified. Here, we show that EGF stimulation induces PKCε-dependent phosphorylation of migration and invasion inhibitory protein (MIIP) at Ser303; this phosphorylation promotes the interaction between MIIP and RelA in the nucleus, by which MIIP prevents histone deacetylase 6 (HDAC6)-mediated RelA deacetylation, and thus enhances transcriptional activity of RelA and facilitates tumor metastasis. Meanwhile PP1, which functions as a phosphatase, is found to mediate MIIP-S303 dephosphorylation and its expression level inversely correlates with metastatic capability of tumor cells. Moreover, clinical analyses indicate the level of MIIP-S303 phosphorylation correlates with colorectal cancer (CRC) metastasis and prognosis. These findings uncover an unidentified mechanism underlying the precise regulation of NF-κB by EGF, and highlight the critical role of nuclear MIIP in tumor metastasis.

## Abstract (web summary)

In colorectal cancer, EGFR signalling is implicated in metastasis. Here, the authors unravel a mechanism through which EGF stimulation induces MIIP phosphorylation, leading to MIIP interacting with RelA—this prevents RelA deactylation and enhances transcriptional activity, facilitating metastasis.

## Introduction

NF-κB signaling pathway is physiologically linked to inflammatory process and is basically involved in the regulation of cellular growth and survival^1, 2^. Its dysregulation has been implicated in the initiation and progression of tumor development^3–5^. The NF-κΒ family is composed of p50 (NF-κB1), p52 (NF-κB2), p65 (RelA), c-Rel, and RelB and functions in the form of heterodimeric and homodimeric complexes^6^. In general, NF-κB is able to be activated via two distinct pathways under various stimuli such like cytokines, growth factors, and oncoproteins. In the canonical pathway, under basal conditions cytoplasmic NF-κΒ binds to their inhibitors IκB. Stimulation of the cell triggers TGFβ-activated kinase 1 (TAK1)-dependent activation of IκB kinase (IKK) complex (IKKα, ΙΚΚβ, and ΙΚΚγ/ΝΕΜΟ)^7^. The IKK activation phosphorylates IκB and promotes its proteasomal degradation, which subsequently leads to nuclear translocation of NF-κB; thereby facilitates the gene transcription of NF-κB-targeted genes^7^. On the other hand, NF-κB activation can be triggered in a non-canonical manner in which NF-κB is cleaved by IKKα, through a process dependent NF-κΒ-inducing kinase (NIK) but ΙΚΚβ and ΙΚΚγ.

The regulation of the NF-κB signal usually becomes more complicated in cross-talking with other cellular signals, as a result its consequent effect is determined in a diverse manner. The cooperative effect between EGFR and NF-κB pathways is importantly implicated in tumourigenesis^8^, among which PKCε signaling has been known involved in EGF-induced NF-κB activation by its direct phosphorylation on IKKβ that eventually leads to RelA activation^9, 10^. As the core signaling transducer of NF-κB pathway, RelA is regulated flexibly with respect to the status of its translational modification including phosphorylation and acetylation^11^. Acetylation in distinct lysine residues affects NF-κB activity differently. For instance, lysine 221 acetylation of RelA selectively enhances its DNA binding while lysine 310 acetylation facilitates its full transcriptional activity independent of regulation of DNA binding or I-κBalpha binding^12^. In turn, acetylated RelA is deacetylated by histone deacetylase 3 (HDAC3). Deacetylation of lysine 221 promotes high-affinity binding of RelA. In this layer, to further study the mechanisms underlying the regulation of RelA activity in the context of EGF/PKCε/NF-κB pathway will be helpful for better understanding the relevant physiological impact.

The migration and invasion inhibitory protein (MIIP) is recognized as a repressor in the regulation of cell growth and invasion^13, 14^. Previous studies indicated MIIP antagonizes insulin-like growth factor binding protein 2 (IGFBP-2)-mediated invasion in glioma cell^15^, and is able to inhibit the enzymatic activity of Histone deacetylase 6 (HDAC6) against α-tubulin acetylation that is related to reduction of cell migration^16^. In addition, MIIP was found to promote EGFR protein degradation and exert the negative effect on proliferation in lung cancer cells^17^. Of note, a recent study suggests nuclear HDAC6 inhibits invasion by suppressing NF-κB/MMP2 signaling^18^. Given on the implication of functional relationship between MIIP and HDAC6, the potential regulatory effect of MIIP on HDAC6 in the nucleus is worthy of investigation to uncover the precise role of MIIP during cell migration and invasion.

Here, we show that activation of EGFR in human cancer cells results in PKCε-dependent MIIP phosphorylation and its interaction with RelA in the nucleus. Intriguingly, phosphorylated MIIP protects deacetylation of RelA from HDAC6, thereby ensures EGFR-stimulated RelA transcriptional activity and potentiates tumor metastasis. Furthermore, PP1 is found to mediate MIIP-S303 dephosphorylation and its downregulation is responsible for the metastatic capability of tumor cells.

## Results

### EGF induces the Interaction between MIIP and RelA

**Fig. 1:**
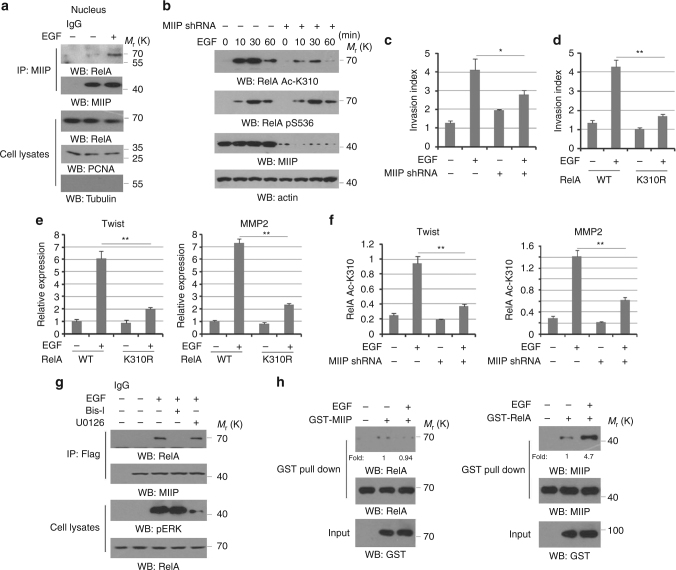
EGF induced the interaction between MIIP and RelA. **a** HCT116 cells were treated with or without EGF. Cellular nucleus-extracts subjected to immunoprecipitation with an anti-MIIP antibody. **b** HCT116 cells transfected with or without plasmid for expressing the indicated MIIP shRNA were treated with or without EGF for indicated periods of time. **c**, **d** HCT116 cells transfected with or without plasmid for expressing MIIP shRNA (**c**), and HCT116 cells expressed with wild type (WT) RelA and RelA K310R (**d**) were treated with or without EGF (100 ng/ml). Cell invasion assays were performed. **e** HCT116 cells expressed with WT RelA and RelA K310R were treated with or without EGF (100 ng/ml) for 10 h. Relative mRNA levels were analyzed by q-PCR. **f** HCT116 cells transfected with or without plasmid for expressing MIIP shRNA were treated with or without EGF (100 ng/ml) for 10 h. ChIP analyses with an anti-RelA Ac-K310 antibody were performed. The primers covering RelA binding site of MMP2 gene promoter region were used for the q-PCR. The *Y* axis shows the value normalized to the input. **g** HCT116 cells were pretreated with Bis-I (2 μM), U0126 (20 μM) for 1 h, prior to EGF treatment (100 ng/ml) for 30 min. Cellular extracts subjected to immunoprecipitation with an anti-Flag antibody. **h** Purified GST-MIIP protein was mixed with mitotic extracts from HCT116 cells treated with EGF (100 ng/ml) for 30 min. GST pull down analyses were performed (left panel). Purified GST–RelA protein was mixed with mitotic extracts from HCT116 cells treated with EGF (100 ng/ml) for 30 min (right panel). In **a**, **b**, **g**, **h**, immunoblotting analyses were performed using the indicated antibodies and data represent one out of three experiments. In **c**–**f**, the values are presented as mean ± s.e.m. (*n* = 3 independent experiments), * represents *P* < 0.05 and ** represents *P* < 0.01 (Student’s *t*-test) between the indicated groups

Based on the vital role of NF-κB signals and MIIP in tumor metastasis, we first examined whether MIIP is involved in the EGF-induced NF-κB activation. Nuclear fraction followed by an immunoprecipitation analysis in HCT116 cells indicated EGF stimulation resulted in a dramatic increase of the interaction between endogenous MIIP and RelA in the nucleus (Fig. 1a and Supplementary Fig. 1a). To investigate the potential effects of nuclear MIIP on NF-κB activity in the level of translational modification, we expressed MIIP shRNAs in HCT116 cells (Supplementary Fig. 1b). Intriguingly, MIIP depletion resulted by according MIIP shRNAs efficiently abrogated EGF-induced RelA acetylation at K310 but not phosphorylation at S536 (Fig. 1b and Supplementary Fig. 1c), both of which are related to RelA transcriptional activity in nucleus^11^. We next examined whether MIIP directly regulates tumor cell invasion and the relevant genes transcription. Consistently with previous reports^16^, transwell analysis of HCT116 cells showed MIIP depletion increased the basal level of cell invasion. However, either EGF-enhanced tumor cell invasion (Fig. 1c) or EGF-induced transcription of MMP2 and Twist (Supplementary Fig. 1d) was compromised by MIIP depletion. These effects were recapitulated in cells with overexpression of acetylation resistant RelA K310R mutant to more profound extents but not its wild type (WT) counterpart (Fig. 1d, e), suggesting the importance of RelA activity in EGF-induced tumor cell invasion. In addition, ChIP analysis showed EGF treatment dramatically increased accumulation of MIIP and RelA AcK310 at promoter regions of MMP2 and Twist genes, which were disrupted by MIIP depletion (Fig. 1f).

PKCε is reported to be responsible for RelA activation upon EGF stimulation^9, 10^. In agreement, PKC inhibitor Bis-I treatment led to impairment of RelA acetylation induced by EGF (Supplementary Fig. 1e). In the meantime, we stably expressed Flag-MIIP in HCT116 cells and found the binding of MIIP to RelA induced by EGF treatment was blocked with PKC inhibition but not treatment with MEK/ERK inhibitor U0126 (Fig. 1g). These results suggest EGF-induced interaction between RelA and MIIP would be attributed to augmented EGFR–PKCε signaling, which thus drive us to further explore the underlying mechanism. As shown in the left panel of Fig. 1h, purified recombinant MIIP protein were incubated with nuclear extracts from HCT116 cells, and GST pull down analysis indicates MIIP has no binding to nuclear RelA regardless of EGF treatment. However, another parallel GST pull down assay showed EGF stimulation significantly promoted the association of nuclear MIIP with purified recombinant RelA protein (Fig. 1h, the right panel). These data suggest the EGF-induced interaction between MIIP and RelA is regulated in a manner dependent on altered status of MIIP but not RelA.

### PKCε phosphorylates MIIP and promotes MIIP–RelA interaction

**Fig. 2:**
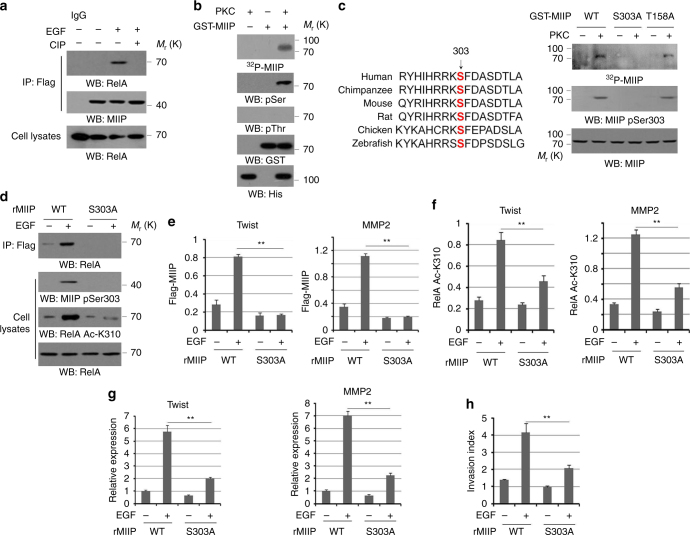
PKCε phosphorylated MIIP and promoted MIIP–RelA interaction. **a** HCT116 cells were treated with or without EGF. Cellular extracts subjected to immunoprecipitation with an anti-Flag antibody. The immunoprecipitates were treated with CIP (10 units), followed by immunoblotting analysis. **b**, **c** In vitro phosphorylation analyses were performed by mixing the purified active PKCε with the indicated purified GST-MIIP proteins in the presence of [γ-32P]ATP. Ser303 of MIIP is evolutionarily conserved in the indicated species (**c**, left panel). **d** HCT116 cells expressed with WT MIIP or MIIP S303A were treated with or without EGF for 30 min. **e** HCT116 cells expressed with WT MIIP or MIIP S303A were treated with or without EGF (100 ng/ml) for 10 h. **f** HCT116 cells with depletion of MIIP, and reconstituted expression of WT rMIIP or rMIIP S303A were treated with or without EGF (100 ng/ml) for 10 h. **g** HCT116 cells with depletion of MIIP, and reconstituted expression of WT rMIIP or rMIIP S303A were treated with or without EGF for 10 h. Relative mRNA levels were analyzed by q-PCR. **h** HCT116 cells with depletion of MIIP, and reconstituted expression of WT rMIIP or rMIIP S303A were treated with or without EGF (100 ng/ml). Cell invasion assays were performed. In **e**, **f**, ChIP analyses with indicated antibodies were performed. The primers covering RelA binding site of MMP2 gene promoter region were used for the q-PCR. The *Y* axis shows the value normalized to the input. In **a**–**d**, immunoblotting analyses were performed using the indicated antibodies. In **e**–**h**, the values are presented as mean ± s.e.m. (*n* = 3 independent experiments), ** represents *P* < 0.01 (Student’s *t*-test) between the indicated groups

We further investigated the key regulatory signals required for the association between MIIP and RelA under EGFR activation. Co-immuoprecipitation analysis showed EGF-induced interaction between MIIP and RelA was abolished in the precipitates incubated with calf intestinal phosphatase (CIP) (Fig. 2a), revealing the interaction is phosphorylation dependent. Along with the results from the GST pull down assay (Fig. 1h), this finding raised the possibility that cellular MIIP might undergo translational modification through EGFR–PKCε pathway. An in vitro protein phosphorylation assay indicated that purified, activative PKCε phosphorylated purified recombinant MIIP and the phosphorylation was detected by immunoblotting analyses only with an anti-phospho-Serine antibody (Fig. 2b), as demonstrated by autoradiography and immunoblotting analysis. In addition, Scansite analysis also showed Ser303 and Thr158 on MIIP are potential PKCε phosphorylated residues, and the autoradiography analysis further showed the mutation of evolutionarily conserved Ser 303 (Fig. 2c, left panel) largely abolished PKCε-mediated MIIP phosphorylation, which is also revealed in an immunoblotting analysis with a specific MIIP-S303 phosphorylation antibody (Fig. 2c, the right panel).

To determine whether PKCε-mediated MIIP-S303 phosphorylation is required for EGF-induced MIIP–RelA interaction, we depleted endogenous MIIP in HCT116 cells and reconstituted the expression of RNAi resistant WT rMIIP and rMIIP S303A (Supplementary Fig. 2a), and the co-immunoprecipitation analysis were performed. In contrast to its Flag-tagged WT counterpart, MIIP S303A mutant which is resistant to MIIP-S303 phosphorylation, largely lost its interaction with RelA under EGF stimulation (Fig. 2d). Meanwhile, we found rMIIP S303A, but not WT rMIIP resulted in compromised enhancement of RelA AcK310 levels induced by EGF stimulation (Fig. 2d). In addition, the effects of MIIP S303A on MIIP–RelA interaction and RelA AcK310 were recapitulated by PKCε depletion (Supplementary Fig. 2b). In accordance with these results, both EGF-induced recruitment of MIIP (Fig. 2e) and accumulated RelA AcK310 (Fig. 2f) at promoter regions of MMP2 and Twist genes were conspicuously blocked in HCT116 cells expressed with rMIIP S303A compared with its WT counterpart. Examination of MMP2 and Twist mRNA levels also showed EGF-induced downstream transcription was compromised by reconstituted expression of rMIIP S303A in HCT116 cells (Fig. 2g), which mirrored (Fig. 1e) but did not aggravate (Supplementary Fig. 2c) the effects from RelA K310R expression. Furthermore, transwell analysis showed rMIIP S303A expression significantly inhibited EGF-induced tumor cell invasion (Fig. 2h), and no additional effects on impaired invasive ability were observed when rMIIP S303A mutant co-expressed with RelA K310R in HCT116 cells compared with its WT counterpart (Supplementary Fig. 2d). Collectively, these results suggest PKCε-mediated MIIP-S303 phosphorylation facilitates the interaction between MIIP and RelA, which is required for EGF-induced tumor cell invasion.

### PP1 mediates MIIP dephosphorylation

**Fig. 3:**
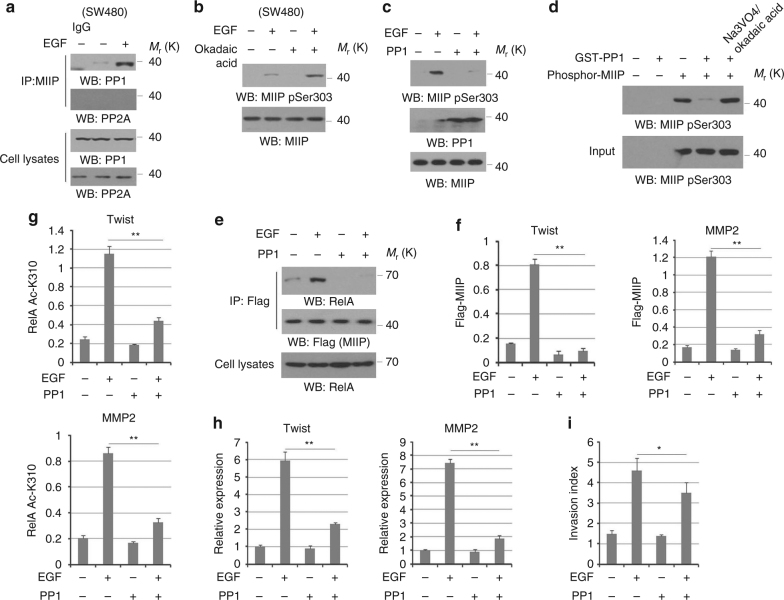
PP1 mediates MIIP dephosphorylation. **a** SW480 cells were treated with or without EGF. Cellular extracts subjected to immunoprecipitation with an anti-MIIP. **b** SW480 cells pretreated with Okadaic acid (a PP1 and PP2A inhibitor) (30 nM) were stimulated with or without EGF (100 ng/ml) for 30 min. **c** HCT116 cells transfected with or without plasmid for expressing PP1 were treated with or without EGF (100 ng/ml) for 30 min. **d** In vitro dephosphorylation analyses were performed by mixing the purified active PP1 with PKCε-phosphorylated GST-MIIP proteins in absence or presence of Na3VO4/Okadaic acid. **e**–**h** HCT116 cells expressing Flag-MIIP were transfected with or without plasmid for expressing PP1. Cells were treated with or without EGF (100 ng/ml) for 10 h. Cellular extracts subjected to immunoprecipitation with an anti-MIIP (**e**). ChIP analyses were performed. The primers covering RelA binding site of Twist or MMP2 gene promoter region were used for the q-PCR (**f**, **g**). Relative mRNA levels were analyzed by q-PCR (**h**). (**i**) HCT116 cells transfected with or without plasmid for expressing PP1 was treated with or without EGF (100 ng/ml). Cell invasion assays were performed. In **f**, **g**, ChIP analyses with indicated antibodies were performed. The primers covering RelA binding site of MMP2 gene promoter region were used for the q-PCR. The *Y* axis shows the value normalized to the input. In **a**–**e**, immunoblotting analyses were performed using the indicated antibodies. In **a**–**e**, immunoblotting analyses were performed using the indicated antibodies and data represent one out of three experiments. In **g**, **f**–**i**, the values are presented as mean ± s.e.m. (*n* = 3 independent experiments), * represents *P* < 0.05 and ** represents *P* < 0.01 (Student’s *t*-test) between the indicated groups

To further evaluate the positive effects of PKCε-mediated MIIP phosphorylation on tumor cell invasion, we examined MIIP-S303 phosphorylation levels in multiple colorectal cancer (CRC) cell lines with distinct invasive capability. Apparently, immunoblotting analysis showed the EGF stimulated stronger MIIP-S303 phosphorylation in CRC cells (Supplementary Fig. 3a) which displayed the higher tendency of invasion (Supplementary Fig. 3b), implying there would be certain cellular component that counteracts PKCε-mediated MIIP phosphoryaltion and restricts invasive ability of tumor cells. The cellular amount of eukaryotic protein phosphorylation is reversibly regulated through phsophatase-mediated dephosphorylation. Phosphoprotein phosphatases (PPP) superfamily includes PP1, PP2A, and is known to participate in diverse biological processes^19^. We wondered that whether MIIP pSer303 is targeted by PP1. Interestingly, co-immuoprecipitation analysis showed the dramatic increased PP1, the catalytic subunit of PP1, but not PP2A was detected in precipitates from MIIP antibody in SW480 cells after EGF stimulation (Fig. 3a). Furthermore, we found either treatment with okadaic acid (OA), an inhibitor for both PP1 and PP2A, markedly enhanced EGF-induced MIIP-S303 phosphorylation in SW480 cells (Fig. 3b), and overexpression of PP1 (Fig. 3c) robustly downregulated MIIP-S303 phosphorylation in HCT116 cells with EGF stimulation. To support these findings, the dephosphorylation assay also showed purified PP1 efficiently abolished MIIP-S303 phosphorylation of purified Flag-MIIP from HCT116 cells, which is reversed by the inhibitors Na3VO4 and OA (Fig. 3d). Thus, these data demonstrate PP1 functions as a physiological MIIP phosphatase.

The effects of PP1 activity on RelA–MIIP complexes were further demonstrated by co-immunoprecipitation and ChIP analyses showing that the interaction between RelA and MIIP (Fig. 3e), as well as the accumulation of MIIP (Fig. 3f) and RelA K310 acetylation (Fig. 3g) at promoter regions of MMP2 and Twist were disrupted in HCT116 cells with overexpression of PP1. Accordingly, transcriptions of MMP2 and Twist were also blocked by PP1 induced by EGF (Fig. 3h). However, transwell analysis showed invasion induced by EGF was attenuated by overexpression of PP1 in HCT116 cells to a lesser extent compared with that effect from MIIP S303A (Fig. 3i), suggesting the additional function of PP1 is implicated in tumor cell invasion. In contrast, PP1 depletion in SW480 cells (Supplementary Fig. 3c) promoted EGF-induced MIIP-S303 phosphorylation as well as RelA–MIIP interaction (Supplementary Fig. 3d), which is accompanied by the dramatically increased RelA K310 acetylation (Supplementary Fig. 3e) and MIIP enrichment at promoters (Supplementary Fig. 3f), and MMP2 and Twist expression levels (Supplementary Fig. 3g).

### MIIP prevents HDAC6-mediated RelA deacetylation

**Fig. 4:**
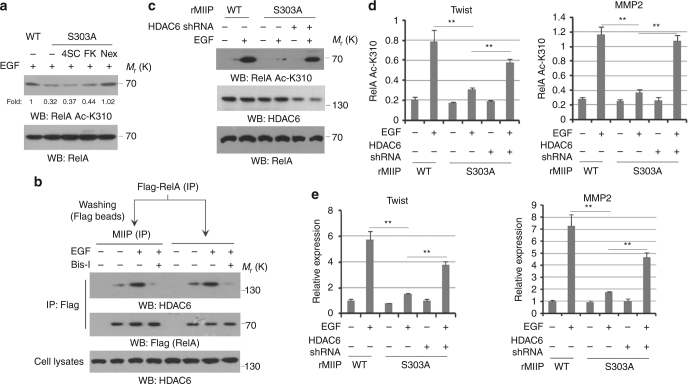
MIIP prevent HDAC6-mediated RelA deacetylation. **a** HCT116 cells were pretreated with or without 4SC-202 (0.6 μM), FK228(20 nM) and Nexturastat A (5 nM) for 1 h prior to EGF (100 ng/ml) treatment for 30 min. Immunoblotting analyses were performed. **b** HCT116 cells were pretreated with or without Bis-l for 1 h prior to EGF (100 ng/ml) treatment for 30 min. Cellular extracts subjected to immunoprecipitation with an anti-Flag, followed by Flag-beads washing and a second immunoprecipitation with an anti-MIIP (lanes 1–4 from left). Cellular extracts subjected to immunoprecipitation with an anti-Flag (lanes 5–8 from left). **c**–**e** HCT116 cells expressed with WT MIIP or MIIP S303A were overexpressed with or without HDAC6 shRNA; cells were treated with or without EGF (100 ng/ml) for 10 h. Immunoblotting analyses were performed (**c**). ChIP analyses with an anti-RelA Ac-K310 antibody were performed. The primers covering RelA binding site of Twist or MMP2 gene promoter region were used for the q-PCR. The *Y* axis shows the value normalized to the input. **d** Relative mRNA levels were analyzed by q-PCR (**e**). In **a**–**c**, immunoblotting analyses were performed using the indicated antibodies and data represent one out of three experiments. In **d**, **e**, the values are presented as mean ± s.e.m. (*n* = 3 independent experiments), ** represents *P* < 0.01 (Student’s *t*-test) between the indicated groups

The effect of MIIP depletion on RelA deacetylation implies MIIP might maintains RelA deacetylation instead of the initial activation (Fig. 1b). To determine this, HCT116 cells were treated with a panel of HDAC inhibitors, and we found MIIP S303A-suppressed RelA K310 acetylation induced by EGF was significantly reversed by the treatment with Nexturastat A, a selective inhibitor against HDAC6, but not 4SC-202 and FK228, which are HDAC class I inhibitors (Fig. 4a). Consistently, MIIP is known to be able to form complex with HDAC6^16^, which is importantly involved in the regulation of RelA acetylation^18^. Hence, we wondered whether MIIP protects RelA acetylation through inhibition of RelA deacetylation by HDAC6. A two step-co-immunoprecipation analysis showed HDAC6 is able to interact with MIIP in precipitates from Flag-RelA in EGF-stimulated HCT116 cells (Fig. 4b, the lanes 1–4), which was abolished by treatment of PKCε inhibitor due to its inhibitory effects on MIIP–RelA interaction. In another co-immunoprecipation analysis by pulling down quantified equal amounts of Flag-RelA from HCT116 cells treated with EGF, we found RelA is associated with HDAC6 (Fig. 4b, the lanes 5–8) to a comparable level to the amount of HDAC6 binding to MIIP after washing precipitates with Flag peptides (Fig. 4b. the lanes 1–4). These data indicated there is a trimmer complex formation in the order of RelA–MIIP–HDAC6, in which MIIP might prevent the direct binding of HDAC6 to RelA and thus protect RelA from HDAC6-mediated deacetylation.

Further, we found MIIP S303A remained to interact with HDAC6 although lost the binding to RelA, and the interaction was not significantly affected by EGF (Supplementary Fig. 4a), revealing MIIP-S303 phosphorylation is only responsible for the complex formation of HDAC6–MIIP–RelA and MIIP might participate in the regulation of HDAC6 function in extra cellular events. Importantly, the downregulation of RelA K310 acetylation resulted by reconstituted expression of rMIIP S303A is blocked by shRNA-mediated depletion of HDAC6 in EGF-stimulated HCT116 cells (Fig. 4c). In accordance, ChIP analyses further showed the impaired accumulation of RelA Ac-310 at promoter regions of MMP2 and Twist resulted by rMIIP S303A was largely reversed in EGF-stimulated HCT116 cells with HDAC6 depletion (Fig. 4d). In accordance, q-PCR analyses showed HDAC6 depletion abolished the inhibitory effects of rMIIP S303A on EGF-induced transcription of MMP2 and Twist (Fig. 4e). These results revealed PKCε-phosphorylated MIIP protects RelA from HDAC6-mediated deacetylation.

KRAS is mutated in HCT116 cells^20^. To clarify whether the regulation of RelA by MIIP is dependent on KRAS status, endogenous MIIP was depleted and the expression of RNAi resistant WT rMIIP and rMIIP S303A were reconstituted in the colon cancer cell line CaCo2 cells (Supplementary Fig. 4b), which express wild type-KRAS. As shown in Supplementary Fig. 4c, EGF readily induced the interaction between RelA and WT MIIP but not MIIP S303A. Similar with that of HCT116, MIIP S303A expression which blocked promoter-enrichment of MIIP (Supplementary Fig. 4d) or RelA Ac-K310 in CaCo2 cells (Supplementary Fig. 4e), inhibited transcription of MMP2 and Twist (Supplementary Fig. 4f) and tumor cell invasion (Supplementary Fig. 4g). Meanwhile, EGF stimulation also led to the trimmer complex formation of RelA–MIIP–HDAC6 in CaCo2 cells (Supplementary Fig. 4h).

### MIIP–RelA facilitates H3-K9 acetylation at promoter region

**Fig. 5:**
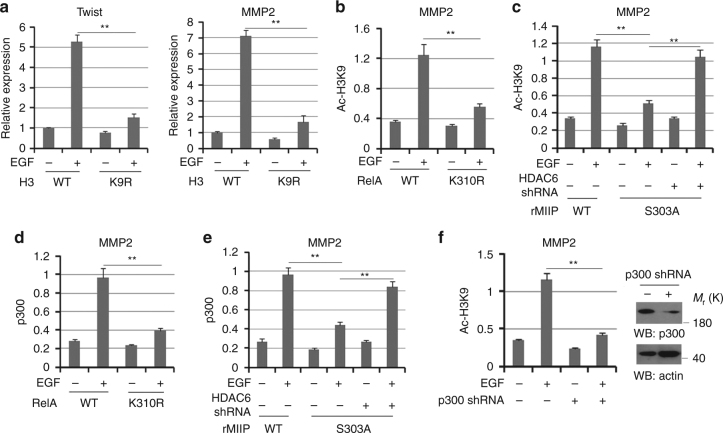
MIIP–RelA facilitates H3-K9 acetylation at promoter region. **a** HCT116 cells expressed with WT H3 or H3 K9R were treated with or without EGF (100 ng/ml) for 10 h. Relative mRNA levels were analyzed by q-PCR. **b**, **d** HCT116 cells expressed with WT RelA or RelA K310R were treated with or without EGF for 10 h (100 ng/ml). **c**, **e** HCT116 cells expressed with WT MIIP or MIIP S303A were overexpressed with or without HDAC6; cells were treated with or without EGF (100 ng/ml) for 10 h. **f** HCT116 cells transfected with or without plasmid for expressing p300 shRNA were treated with or without EGF (100 ng/ml) for 10 h. In **b**–**f**, ChIP analyses with indicated antibodies were performed. The primers covering RelA binding site of MMP2 gene promoter region were used for the q-PCR. The *Y* axis shows the value normalized to the input. In **a**–**f**, the values are presented as mean ± s.e.m. (*n* = 3 independent experiments), ** represents *P* < 0.01 (Student’s *t*-test) between the indicated groups

Increased K310 acetylation of RelA promotes its transcriptional activity for the downstream genes expression and is usually linked to the regulation of H3 acetylation or methylation at genes promoter region^21–23^. Time course analysis of various histone marks for transcription indicated accumulation of Ac-H3K9 and H3K4me3 but not Ac-H3K18 or H3K36Me2 at the MMP2 promoter region were notably increased in HCT116 cells with EGF stimulation (Supplementary Fig. 5a). Subsequently, HCT116 cells were overexpressed with WT H3 or H3 K9R mutant, in which the lysine was mutated into arginine (Supplementary Fig. 5b). ChIP analysis showed EGF stimulated a dramatic increase of H3-K9 acetylation at MMP2 promoter region, which was abolished by H3 K9R (Supplementary Fig. 5c). In the meantime, q-PCR analyses showed EGF-induced upregulation of Twist and MMP2 were tremendously inhibited by H3 K9R expression (Fig. 5a). We next examined whether MIIP–RelA interaction at MMP2 promoter region is involved in the regulation of histone H3 acetylation. As a result, EGF-induced H3-K9 acetylation at MMP2 promoter was blocked either by RelA K310R expression (Fig. 5b), or rMIIP S303A (Fig. 5c), compared with their WT counterparts. Additionally, we found rMIIP S303A induced downregulation of histone H3-K9 acetylation at MMP2 promoter was inhibited in HCT116 cells with HDAC6 depletion (Fig. 5c). In addition, H3K9R overexpression largely blocked EGF-induced H3K4me3 at the MMP2 promoter region (Supplementary Fig. 5d), suggesting the prerequisite role of Ac-H3K9 for H3K4me3 induction. Accordingly, overexpression of Rel K310R or reconstituted expression of rMIIP S303A, which inhibited promoter-associated Ac-H3K9 (Fig. 5b, c), led to decreased H3K4me3 enrichment at promoter (Supplementary Fig. 5e, f). ChIP analysis further indicated the negative effects of rMIIP S303A on H3K4me3 was reversed by HDAC6 depletion (Supplementary Fig. 5f). All together, these data suggest Ac-H3K9 and H3K4me3 are induced by EGF in a sequential manner and MIIP-mediated maintenance of RelA acetylation was critical for the subsequent accumulation of H3-K9 acetylation at promoter region.

To further investigate the mechanism underlying MIIP–RelA-regulated histone H3 acetylation, we performed ChIP analyses with antibodies against p300, a well-known acetyltransferase responsible for H3-K9 acetylation^24, 25^. Consequently, EGF treatment promoted p300 accumulation at MMP2 promoter in HCT116 cells, which was inhibited by overexpression of RelA K310R (Fig. 5d) or MIIP S303A (Fig. 5e), compared with their WT counterparts. Consistent with its effects on H3 lysine9 acetylation, HDAC6 depletion restored p300 accumulation at MMP2 promoter in HCT116 cells with expression of MIIP S303A (Fig. 5e). Importantly, depletion of p300 largely abolished EGF-induced H3-K9 acetylation at MMP2 promoter region in HCT116 cells (Fig. 5f). These results suggest MIIP–RelA interaction is required for the H3-K9 acetylation mediated by p300 at promoter region.

### MIIP-S303 phosphorylation is required for tumor metastasis

**Fig. 6:**
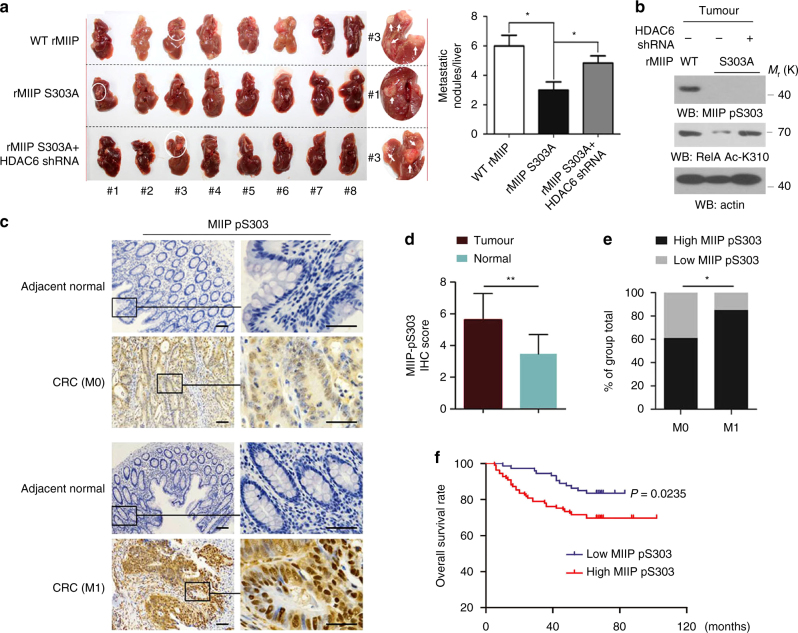
MIIP-S303 phosphorylation is required for tumor metastasis and is related to poor prognosis in human colorectal cancer. **a** SW620 cells with reconstituted expression of WT rMIIP, rMIIP S303A and rMIIP S303A/HDAC6 shRNA were intraspleenically injected into the athymic nude mice. Representative tumor xenografts were shown (left panel). The number of visible metastatic lesions in the liver was measured. Data represent the means±s.e.m. (*n* = 8, right panel), * represents *P* < 0.05 (Student’s *t*-test). **b** Lysates obtained from the pool of tumor tissues (*n* = 8) developed from SW620 cells (as indicated in Fig. 6a) were subjected to immunoblotting analyses using the indicated antibodies. **c** Immunohistochemical staining with anti-MIIP pS303 was performed on 182 human colorectal cancer specimens. Representative photos of non-metastasis and metastasis tumor versus the adjacent normal tissues were shown (magnification: ×100 and ×400). Scale bars: 100 μm. **d** MIIP pS303 levels in tumor and the adjacent normal tissues of colorectal cancer. **e** MIIP pS303 levels in tumor metastasis (M1) or non-metastasis subgroups (M0). The Chi-square test indicating the tight correlation between MIIP pS303 levels and tumor metastasis. **f** The survival times for 182 patients with low (score < 6, blue curve) versus high ((score ≥ 6, red curve) MIIP pS303 levels (low, 73 patients; high, 109 patients). The Kaplan–Meier method and log-rank tests indicating the significant association of MIIP pS303 levels (P = 0.0235) with patient survival. In **a**, **d**, * represents *P* < 0.05 and ** represents *P* < 0.01 (Student’s *t*-test) between indicated groups

To determine whether MIIP-S303 phosphorylation promotes colorectal cancer cell metastasis, SW620 cells were reconstituted with expression of WT rMIIP, rMIIP S303A and rMIIP S303A/HDAC6 shRNA (Supplementary Fig. 6a). EGF treatment in SW620 cells notably induced the interaction between WT MIIP and RelA but not MIIP S303A, and increased RelA Ac-K310 levels in cells with reconstituted expression of WT rMIIP instead of rMIIP S303A (Supplementary Fig. 6b). In addition, EGF notably promoted HDAC6–MIIP–RelA complex formation in a PKCε activation-dependent manner (Supplementary Fig. 6c). Then a liver metastasis mouse model was constructed via intraspleenic injection (Fig. 6a, left panel). Consequently, tumor cells expressed with WT MIIP exhibited the strong capability of metastasis. In contrast, expression of MIIP S303A dramatically inhibited liver metastasis (Fig. 6a, right panel). However, the impaired liver metastasis by MIIP S303A expression was significantly reversed by HDAC6 depletion (Fig. 6a, right panel). In accordance, immunoblotting analysis of mice tumor tissues indicated WT rMIIP but not rMIIP S303A underwent S303 phosphorylation during tumor growth, and RelA Ac-K310 levels was attenuated in tumor tissues from cells with rMIIP S303A expression in a HDAC6 dependent manner (Fig. 6b). Collectively, these results suggest MIIP-S303 phosphorylation maintains RelA Ac-K310 levels in colon cancer cells in vivo through its antagonistic effects on HDAC6, thus facilitates tumor metastasis.

To examine the clinical relevance of the PKCε-mediated MIIP-S303 phosphorylation, we performed IHC-staining analyses in 182 serial sections of human colorectal cancer patient specimens (Fig. 6c and Supplementary Table 1). As a result, MIIP-S303 phosphorylation was increased in colorectal cancer cells compared with adjacent normal colorectal cells (Fig. 6c, d). Moreover, MIIP-S303 phosphorylation level was significantly correlated with colorectal cancer metastasis (*P* = 0.028; Fig. 6c, e). Notably, patients whose tumor expressed a high level (score ≥ 6) of MIIP-S303 phosphorylation had significantly shorter overall survival than those whose tumor expressed no or a low level (score < 6) of MIIP-S303 phosphorylation (Fig. 6f). Furthermore, immunoblotting analysis of tumor tissues of clinical samples indicated MIIP S303 phosphorylation inversely correlates with PP1 levels (Supplementary Fig. 6d, e, upper panel); however, we found MIIP pS303 levels in tumor tissues were not correlated with total MIIP levels (Supplementary Fig. 6d, e, bottom panel), and MIIP levels even dropped in certain proportion of tumor tissues compared with the normal counterparts (Supplementary Fig. 6d). These results demonstrated the importantly indicative role of PKCε-dependent MIIP-S303 phosphorylation in clinical behavior of human colorectal cancer.

## Discussion

**Fig. 7:**
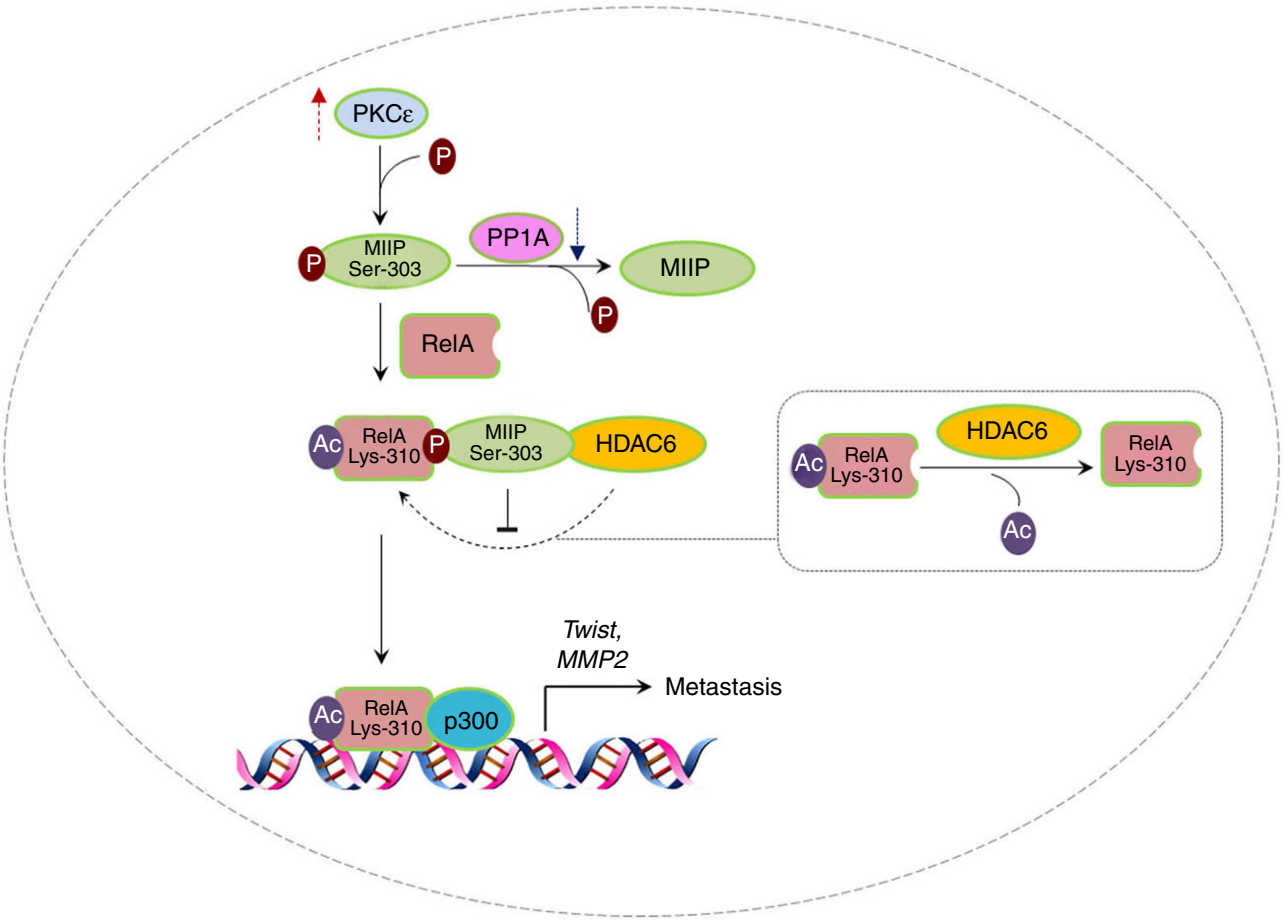
A schematic diagram of model displaying the implication of MIIP in PKCε activation-induced tumor metastasis. PKCε phosphorylates MIIP at Ser303 and promotes its interaction with RelA, which protects RelA from deacetylation by HDAC6; in turn, this leads to enhancement of RelA transcriptional activity and increase of genes expression relevant to cell invasion. PP1 acts as a phosphotase being able to dephosphorylate MIIP phosphorylation by PKCε. In cancer cells, PKCε activity is hyperactive while PP1 protein levels is downregulated; thereby, PKCε /MIIP/RelA signaling is reinforced and faciliates tumor metastasis

MIIP is identified as a suppressor in cell migration and invasion by its inhibitory effects on the relevant effectors such like IGFBP-2 and HDAC6^13^, yet how the functional status of MIIP is regulated remains unclear. In the present study, we show PKCε phosphorylates MIIP at Ser303 and promotes its binding to RelA; in turn, MIIP prevent RelA deacetylation from HDAC6 and facilitates RelA K310 acetylation-dependent expression of downstream genes including Twist, MMP2, thus promotes the cell capabilities of invasion (Fig. 7). These results illustrate a novel regulatory mechanism underlying the effect of MIIP on cell migration and invasion under the activation of EGF–PKCε signaling.

Expression levels of MIIP inversely correlate with tumor malignity^17, 26–29^. Consistent with previous reports^26^, we found interference of overall MIIP expression results in enhanced invasion basally in CRC cell, and MIIP levels are downregulated in tumor tissues of patients with stronger metastatic tendency. However, our study also shows MIIP depletion slightly suppresses the cell invasion instead under the condition of EGF activation (Fig. 1b); and replacement of WT MIIP with MIIP S303A mutant (the mutant resistant to PKCε phosphorylation) dramatically inhibit EGF-induced cell invasion. Thus, these results indicate MIIP are switched into a driving force for cell invasion by PKCε-mediated phosphorylation, and this fraction might play a dominative role under EGF stimulation compared with the rest pool of MIIP that exerts negative effects on cell invasion. Furthermore, clinical analysis shows MIIP Ser303 phosophorylation levels positively correlate the poor prognosis in colorectal cancer patients, which suggests MIIP-regulated invasion in tumor cell is not only coupled with its expression level but also its phosophorylation status if EGF–PKCε signaling is taken into account. Of note, it does not mean our results contradict the established function of MIIP in concern of cell motility, as in previous studies the signaling context is not restricted^16, 17^. In this regard, it can be assumed that MIIP Ser303 phosophorylation would become a decisive factor for malignity progression in tumors with well-expressed MIIP.

PP1 is a serine/threonine specific protein phosphatase and deficiency of its function has been implicated in tumourigenesis of CRC^19, 30–32^. In our study, MIIP Ser303 phosphorylation is identified as a substrate of PP1. MIIP Ser303 phosphorylation displays an inverse relationship with PP1 protein levels in cancer cells. Therefore, MIIP Ser303 phosphorylation is also circumstantially regulated in a manner dependent on PP1 expression level, in addition to PKCε activity; given all this, it will be difficult to precisely evaluate MIIP function on cell invasion unless whole genetic background related is clearly defined.

MIIP is known to block cell migration through its inhibitory effects on the deacytalse activity of HDAC6 against alpha-tubulin^16^. With respect to HDAC6, it is also able to inhibit cancer cell migration by removing RelA acetylation^18^. Thus the effect of HDAC6 on cell migration is context dependent. Our study shows MIIP with Ser303 phosphorylation potentiates cell invasion through its interaction with RelA, which can be explained by its concomitant binding to HDAC6 as the trimmer formed by RelA/MIIP/HDAC6 is detected. Hence, MIIP negatively regulates HDAC6 in distinct signaling environmental conditions, while leads to contrary physiological outcome. Particularly, MIIP/RelA complex is exclusively distributed in the nucleus despite MIIP Ser303 phosphorylation can be detected in the cytosol; this reveals the function of MIIP with respect to cell migration and invasion is likely to be separated based on its subcellular localization; the access of cytosolic MIIP to RelA might be prevented by the abundant RelA-associated component in the cytosol.

Cancer invasion is associated with the abnormal regulation of components responsible for cell motility. The illustration of the mechanism by which MIIP inhibits RelA deacetylation under EGF activation sheds instrumental insight into how cell motility is subtly regulated in response to environmental stimulus; this finding importantly provides a molecular basis for developing therapy of malignant tumors with upregulated PKCε/MIIP/RelA signaling.

## Methods

### Cell culture

HCT116 cells were cultured in McCoy’s 5A medium (Invitrogen) supplemented with 10% FBS. CaCo2 cells were cultured in minimum essential medium (MEM, Invitrogen) supplemented with 10% FBS. SW1116, SW480 and SW620 cells were cultured in Leibovitz’s L-15 medium (Invitrogen) supplemented with 10% FBS. All of the cell lines used in this study were obtained from ATCC and routinely tested for mycoplasma contamination. No cell lines used in this study were found in the database of commonly misidentified cell lines that is maintained by ICLAC and NCBI Biosample. The cell lines were not authenticated.

### DNA constructs and mutagenesis

The pGIPZ human MIIP shRNA was generated with the oligonucleotides 5′-GCTACAACTCAGAGACTCC-3′ for clone 1, 5′-AGGAAGACCATGAATGCGT-3′ for clone 2, and 5′-CTGAGTTGTAGCTGCTGCT-3′ for clone 3. pGIPZ human PKCε was generated with the oligonucleotide 5′-CAAGTTCGGTATCCACAAC-3′ and 5′-AGTTCATGGCCACCTATCT-3′ shRNA pGIPZ human PP1 shRNA was generated with the oligonucleotide 5′-TCGTAGAAACCATAGATGC-3′ and 5′-CCAGGTTGAGCTTCTCGCT-3′. pGIPZ human p300 shRNA was generated with the oligonucleotide 5′-CTAGAGACACCTTGTAGTA-3′. pGIPZ human HDAC6 shRNAs were generated with the oligonucleotides 5′-TTCAGGATGGGATGCAGTG-3′. The pGIPZ controls were generated with control oligonucleotide 5′-GCTTCTAACACCGGAGGTCTT-3′ or 5′-GCCCGAAAGGGTTCCAGCTTA-3′.

### Materials

Antibodies that recognize PP1 (ab137512, 1:1000), PP2A (ab32141, 1:1000), Ac-RelAK310 (ab19870, 1:1000), Ac-H3K9 (ab10812, 1:1000), Ac-H3K18 (ab1191, 1:1000), H3K36Me2 (ab9049, H3K4Me3 (ab8580, 1:1000), and H3 (ab1791, 1:1000) were purchased from Abcam. Antibodies that recognize Flag (#35535, 1:1000), Myc (#T504, 1:1000), His (#T505, 1:1000), an GST (#T509, 1:1000) were obtained from Signalway Antibody. Antibodies against β-actin (#4970, 1:2000), p-Acc (#11818, 1:500), RelA (#8242, 1:1000), and RelA pS536 (#8214, 1:500) were purchased from Cell Signaling Technology. The antibody that recognize MIIP (HPA044948, 1:1000) were purchased from Sigma-Aldrich. Rabbit polyclonal MIIP pSer-303 antibody (1:500) was made by Signalway Antibody. A peptide containing MIIP pSer-303 antibody was injected into rabbits. The rabbit serum was collected and purified using an affinity column with non-phosphorylated MIIP Ser-303 peptide to exclude the antibodies for non-phosphorylated MIIP, followed by an affinity column with phosphorylated MIIP Ser-303 peptide to bind to and purify the MIIP pSer-303 antibody. The MIIP pSer-303 antibody was then eluted and concentrated.

Bis-l was purchased from Cell Signaling Technology (Boston, USA). EGF and U0126 were purchased from Sigma-Aldrich (St. Louis, MO). PKCε was from GeneTex (Irvine, USA). BrdU were purchased from Sigma-Aldrich (St. Louis, USA). OGT and OGA were ordered from R&D (Minneapolis, USA). Nexturastat A, 4SC-202, and FK228 were from Selleckchem (Houston, USA).

### Immunoprecipitation and immunoblotting analysis

Proteins were extracted from cultured cells using a modified buffer, followed by immunoprecipitation and immunoblotting with the corresponding antibodies. Proteins were extracted from cultured cells using a modified buffer (50 mM Tris-HCl (pH 7.5), 1% Triton X-100, 150 mM NaCl, 0.5 mM EDTA, 1 mM dithiothreitol (DTT), and protease inhibitor cocktail or phosphatase inhibitor cocktail), followed by immunoprecipitation and immunoblotting with the corresponding antibodies. The protein concentration was determined through Bradford assay. Proteins from cellular lysates or nuclear extracts were separated by SDS-PAGE, transferred onto PVDF membrane (Millipore Corporation) and probed with the indicated antibodies. Unprocessed original scans of blots are shown in Supplementary Fig. 7.

### Recombinant protein purification

WT and mutant GST-MIIP were expressed in bacteria and purified. Briefly, constructs were used to transform BL21/DE3 bacteria. The cultures were grown at 37 °C to the OD 600 nm of ∼0.6 before isopropyl-β-d-thiogalactopyranoside treatment for 3 h. Cell pellets were collected and lysed by sonication. For GST-tagged proteins, cleared lysates were bound to glutathione-agarose. For His-tagged proteins, cleared lysates were bound to Talon metal affinity resin. Eluates were concentrated using Ultrafree-15 centrifugal filters (Millipore).

### Gene expression analysis

We isolated total RNA from cells using RNAzol RT (Molecular Research Center) following the manufacturer’s instructions. We synthesized cDNA from 1 μg total RNA using iScript cDNA synthesis kit (Bio-Rad) and quantified mRNA levels by real-time qRT-PCR using SYBR Green (Bio-Rad). We ran samples in technical triplicates and calculated relative mRNA levels normalized to actin mRNA levels in the same samples. The qPCR primer sequences were listed: human MMP2′: 5′-CCACTGCCTTCGATACAC-3′ (forward) and 5′-GAGCCACTCTCTGGAATCTTAA-3′ (reverse); human Twist: 5′-ACAAGCTGAGCAAGATTCAGACC-3′ (forward) and 5′-TCCAGACCGAGAAGGCGTAG-3′ (reverse).

### In vitro kinase assay

Purified WT and mutant GST-MIIP were incubated with PKCε in kinase assay buffer supplemented with 0.2 mM AMP and cold ATP in the presence or absence of 0.2 mCi/ml hot ATP (ICN Biochemicals) for 20 min at 30 °C. After the reaction, PKCε was removed by extensive washing with RIPA buffer and kinase assay buffer. The GST-MIIP bound beads were recovered by centrifugation. For kinase phosphorylation analyses, the GST-MIIP bound beads were subjected to SDS-PAGE and then autoradiography after incubation with EN3HANCE (PerkinElmer). The GST-MIIP bound beads after PKCε treatment in absence of hot ATP were further incubate with cellular extracts as indicated.

### ChIP assay

A ChIP assay was performed using an Upstate Biotechnology kit. Quantitative real-time PCR was used to measure the amount of bound DNA, and the value of enrichment was calculated according to the relative amount of input and the ratio to IgG. The primers covering RelA binding site of human Twist1 gene promoter region were used for the real-time PCR: 5′-TTTGGGAGGACGAATTGTTAGACC-3′ (forward) and 5′-TGGGCGAGAGCTGCAGACTTGG-3′ (reverse); and the primers covering RelA binding site of human MMP2 gene promoter region were used for the real-time PCR: gene promoter region were used for the real-time PCR: 5′-CCACTGCCTTCGATACAC-3′ (forward) and Reverse: 5′-GAGCCACTCTCTGGAATCTTAAA-3′ (reverse).

### Nuclear and cytoplasmic extracts

Cellular fractionation was performed using a Nuclear and Cytoplasmic Protein Extraction Kit (Beyotime Biotechnology, Shanghai, China). Briefly, the cells were harvested in ice-cold PBS and suspended in the hypotonic buffer with incubation for 15 min on ice. After vortex for 5 s, the detergent was added into cells and incubated for 1 min on ice, followed by centrifugation at 16,000 × *g* for 5 min at 4 °C. The cytoplasmic fraction was transfered into separate tubes. The nuclear fraction was lysed in the complete lysis buffer on ice for 30 min and centrifuged at 16,000 × *g* for 10 min at 4 °C.

### Cell invasion assay

Cells were seeded in 24-well invasion chambers (BD Biosciences, San Jose, CA, USA) with the Matrigel-coated film insert (8 mm pore). The mixed solution was diluted to give a 1× DMEM solution containing 10% serum. The cells were seeded in absence or presence of EGF (100 ng/ml) (chemokinesis). Two days later, cells on the bottom surface of the filter subjected to staining with 4′,6-diamidino-2-phenylindole for 1 min, then washed three times with PBS, and the cell number was counted under a fluorescence microscope (Olympus).

### Human tissue specimens and immunohistochemical analysis

Human tumor samples and their paired noncancerous matched tissues were obtained from 182 CRC patients treated at the hospital between 2008 and 2011. Written informed consent was obtained from each patient and the investigation was approved by the institutional review board of Zhongshan Hospital, Fudan University, Shanghai, China. Patients with radiotherapy or chemotherapy treatment before surgery were excluded. Survival time was calculated from the date of surgery to the date of death or last follow-up. Clinical information was collected from the medical records of each patient and shown in Supplementary Table 1. The tumor-node-metastasis (TNM) staging was performed according to American Joint Committee on Cancer (AJCC) standards.

Consecutive sections of formalin-fixed, paraffin-embedded (FFPE) tumors were subjected to IHC analysis for MIIP pS303. Rabbit polyclonal MIIP pSer-303 antibody (Signalway, 1:50) was used. A DAB substrate kit (GTVision Detection System/Mo&Rb Kit) was used according to manufacturer’s instructions. The results were scored by two pathologists blinded to the clinicopathologic data.

### Mouse

All animal experiments were approved by the animal care and use committee of Fudan University. Twenty-four female Balb/c nude mice (5-week-old) were divided into three groups (eight mice per group): SW620 cells were reconstituted with expression of WT rMIIP, rMIIP S303A, and rMIIP S303A/HDAC6 shRNA. A small left abdominal flank incision was made, the spleen was exteriorized, and the prepared cells (2 × 10^6^ cells/50 µl/mouse) were injected into the spleen with a 30-Gauge needle. To prevent tumor cell leakage and bleeding, a cotton swab was held over the site of injection for 1 min. The injected spleen was returned to the abdomen and the wound was sutured with 6-0 black silk. Six weeks later, all of the mice were sacrificed and necropsied for observation of visible metastatic lesions in the liver.

### Statistical analysis

Differences between groups were calculated using the Student *t*-test, chi-square test, or the Fisher exact test. The log-rank test was used to obtain a *P*-value for the significance of divergence of Kaplan–Meier curves. All probability values were two-sided. Analyses were performed with the SPSS version 22.0 and Graphpad Prism 6.02 statistical analysis software. Statistical significance was defined as *P* < 0.05.

### Data availability

All relevant data supporting the findings of this study are available from the corresponding author on reasonable request.

## Electronic supplementary material

Supplementary Information
