# Supplementary material for: PKCε phosphorylates MIIP and promotes colorectal cancer metastasis through inhibition of RelA deacetylation
Source: Nat Commun. 2017 Oct 16;8:939. doi: 10.1038/s41467-017-01024-2 (PMC5643311; doi:10.1038/s41467-017-01024-2)
Supplement: Supplementary file 1 — Supplementary Information [file 41467_2017_1024_MOESM1_ESM.pdf]

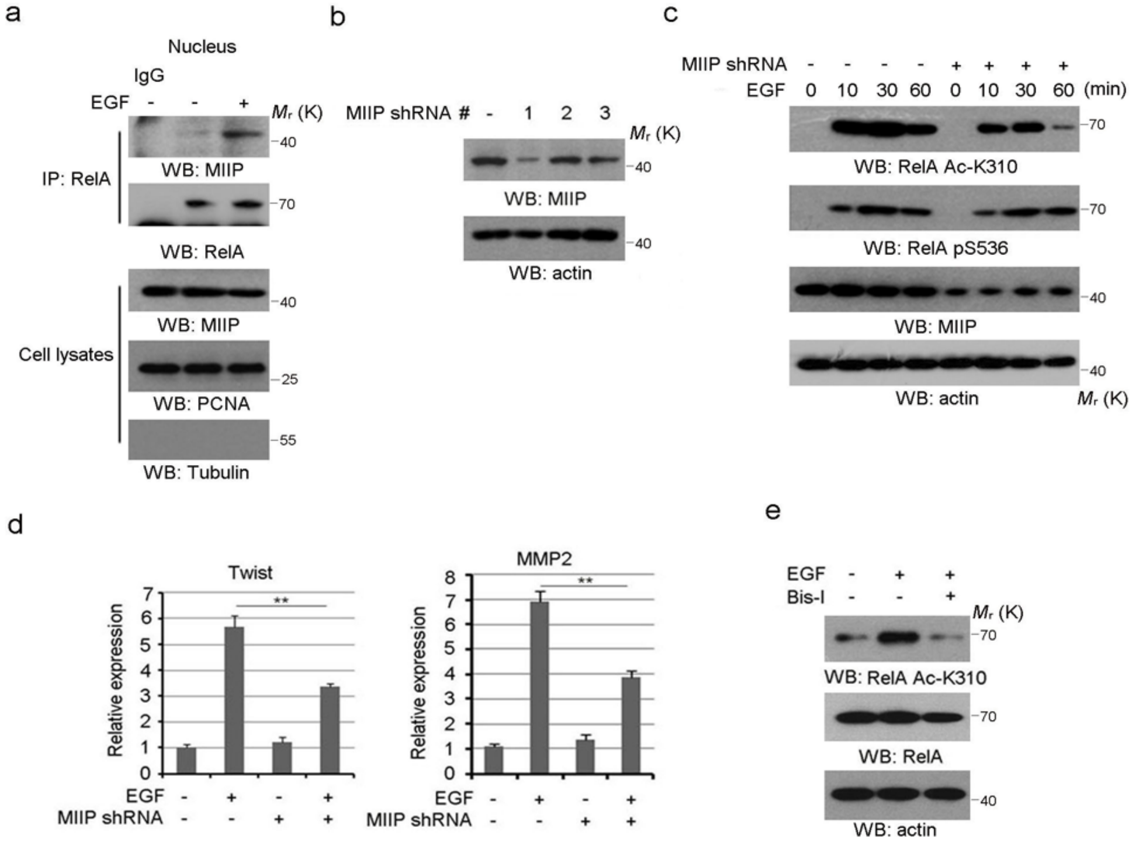

Supplementary Figure 1. EGF induced the interaction between MIIP and RelA. **(a)** HCT116 cells were treated with or without EGF. Cellular nucleus-extracts subjected to immunoprecipitation with an anti-RelA antibody. **(b)** HCT116 cells were transfected with or without plasmid for expressing MIIP shRNA. Immunoblotting analyses were performed using the indicated antibodies. Data represent 1 out of 3 experiments. **(c)** HCT116 cells transfected with or without plasmid for expressing indicated MIIP shRNA were treated with or without EGF for indicated periods of time. Immunoblotting analyses were performed using the indicated antibodies. **(d)** HCT116 cells transfected with or without plasmid for expressing MIIP shRNA were treated with or without EGF for 30 min. Relative mRNA levels were analyzed by q-PCR, the values are presented as mean ± s.e.m. (n = 3 independent experiments). ** represents P<0.01 (Student's *t*-test) between indicated groups. **(e)** HCT116 cells were pretreated with Bis-l (10μM), for 1 h, prior to EGF treatment for 30 min. In a-c and e, immunoblotting analyses were performed using the indicated antibodies. Data

represent 1 out of 3 experiments.

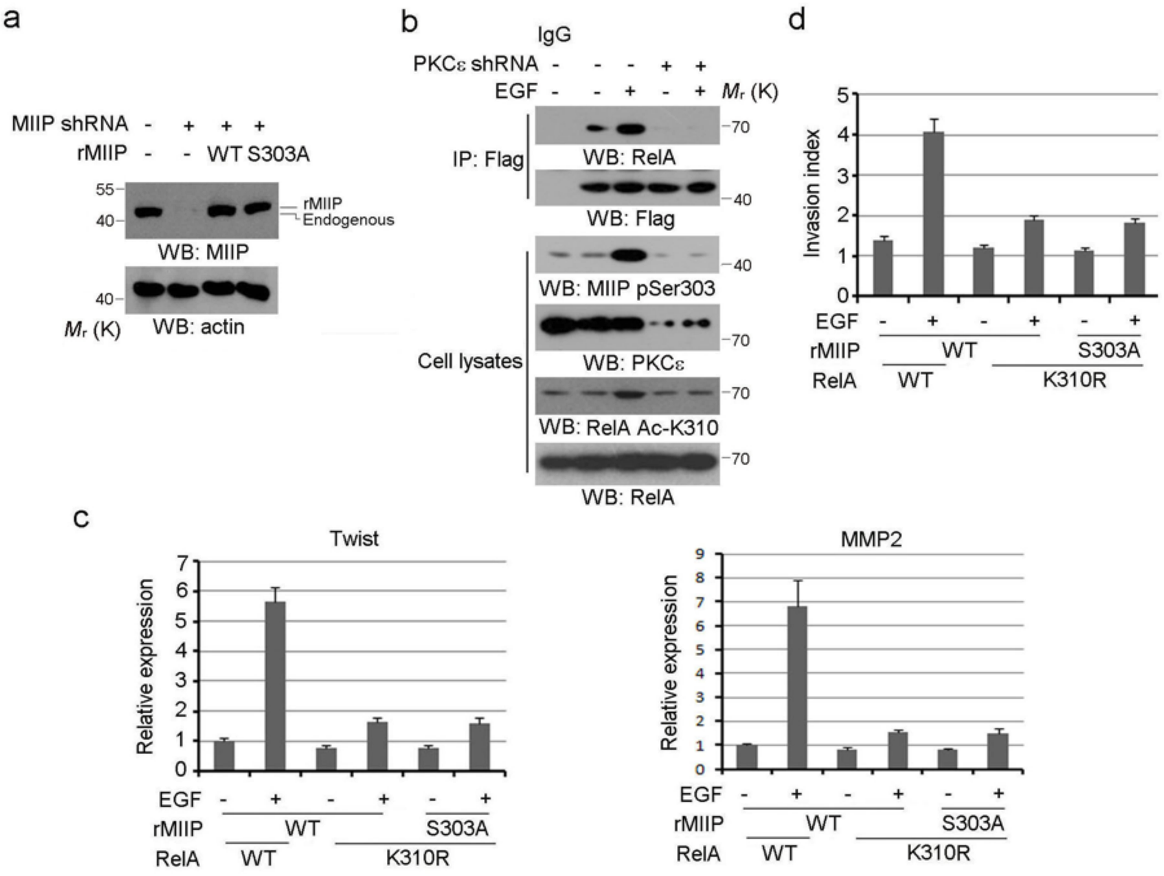

Supplementary Figure 2. PKCε phosphorylated MIIP and promoted MIIP-RelA interaction. (a) HCT116 cells were expressed with a vector for control shRNA or MIIP shRNA and reconstituted with expression of WT rMIIP or rMIIP S303A. **(b)** HCT116 cells expressing Flag-MIIP were transfected with or without plasmid for expressing PKCε shRNA and treated with or without EGF for 30 min. Cellular extracts subjected to immunoprecipitation with an anti-Flag. (c and d) HCT116 cells with depletion of MIIP, and reconstituted expression of WT rMIIP or rMIIP S303A were expressed with WT RelA or RelA K310R. Cells were treated with or without EGF for 30min. Relative mRNA levels were analyzed by q-PCR (c). Cell invasion assays were performed (d). In a and b, immunoblotting analyses were performed using the indicated antibodies. Data represent 1 out of 3 experiments. In c and d, the values are presented as mean ± s.e.m. (n=3 independent experiments).

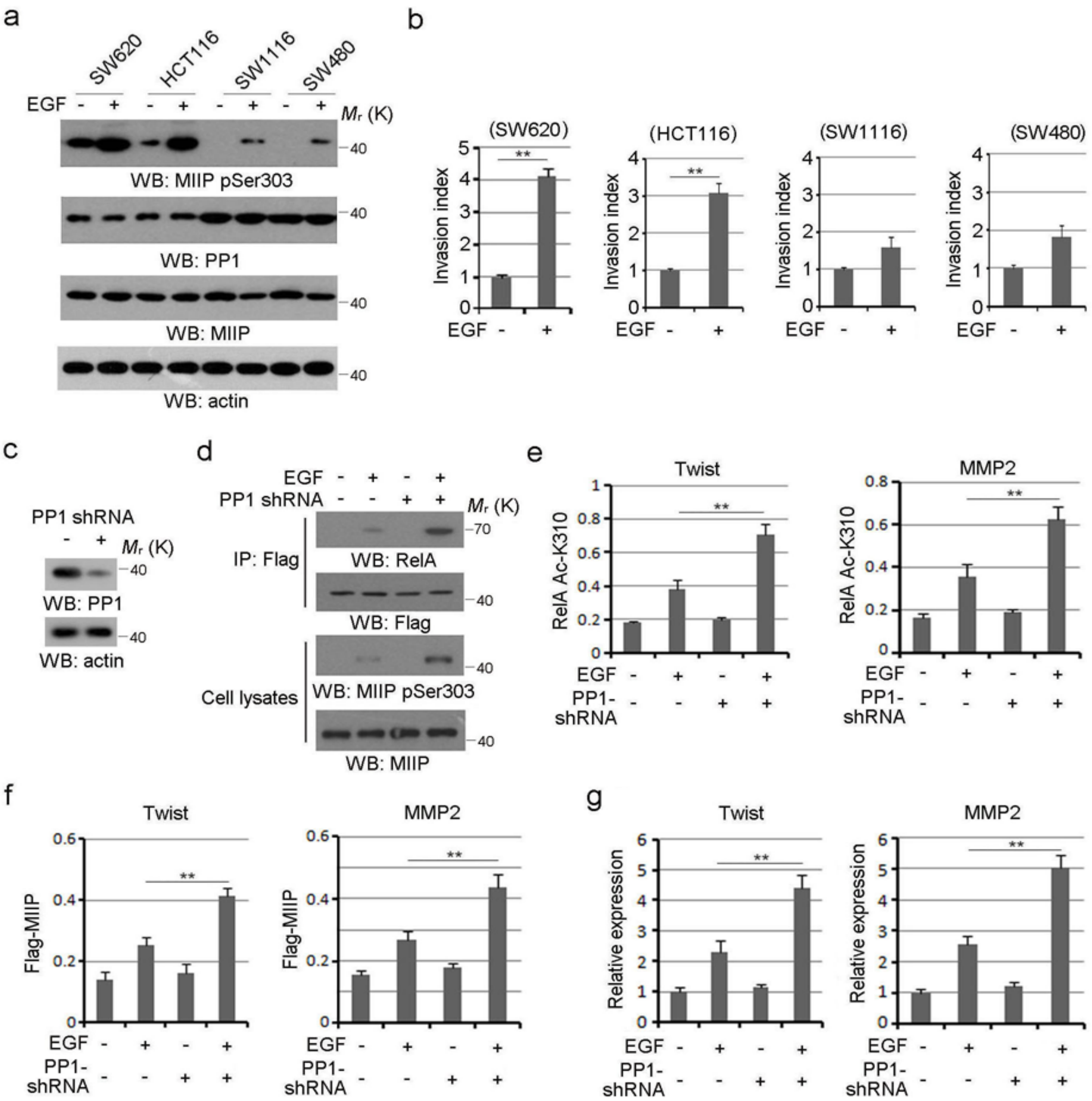

Supplementary Figure 3. PP1 mediates MIIP dephosphorylation. (a and b) Cells of the indicated cell lines were treated with or without EGF for 30min. immunoblotting analyses were performed using the indicated antibodies. Data represent 1 out of 3 experiments (a). Cell invasion assays were performed, the values are presented as mean ± s.e.m. (n = 3 independent experiments). ** represents *P*<0.01 between indicated groups (b). (**c-g**) HCT116 cells expressing Flag-MIIP were transfected with or without plasmid for expressing PP1 shRNA (c). Cells were treated with or without EGF (100 ng/ml) for 10 h. Cellular extracts subjected to immunoprecipitation with an anti-Flag (d). ChIP analyses were performed. The primers covering RelA binding site of Twist or MMP2 gene promoter region were

used for the q-PCR (e, f). Relative mRNA levels were analyzed by q-PCR (g). In a, c and d, immunoblotting analyses were performed using the indicated antibodies. In b, e-g, the values are presented as mean ± s.e.m. (n=3 independent experiments), ** represents *P*<0.01 between the indicated groups.

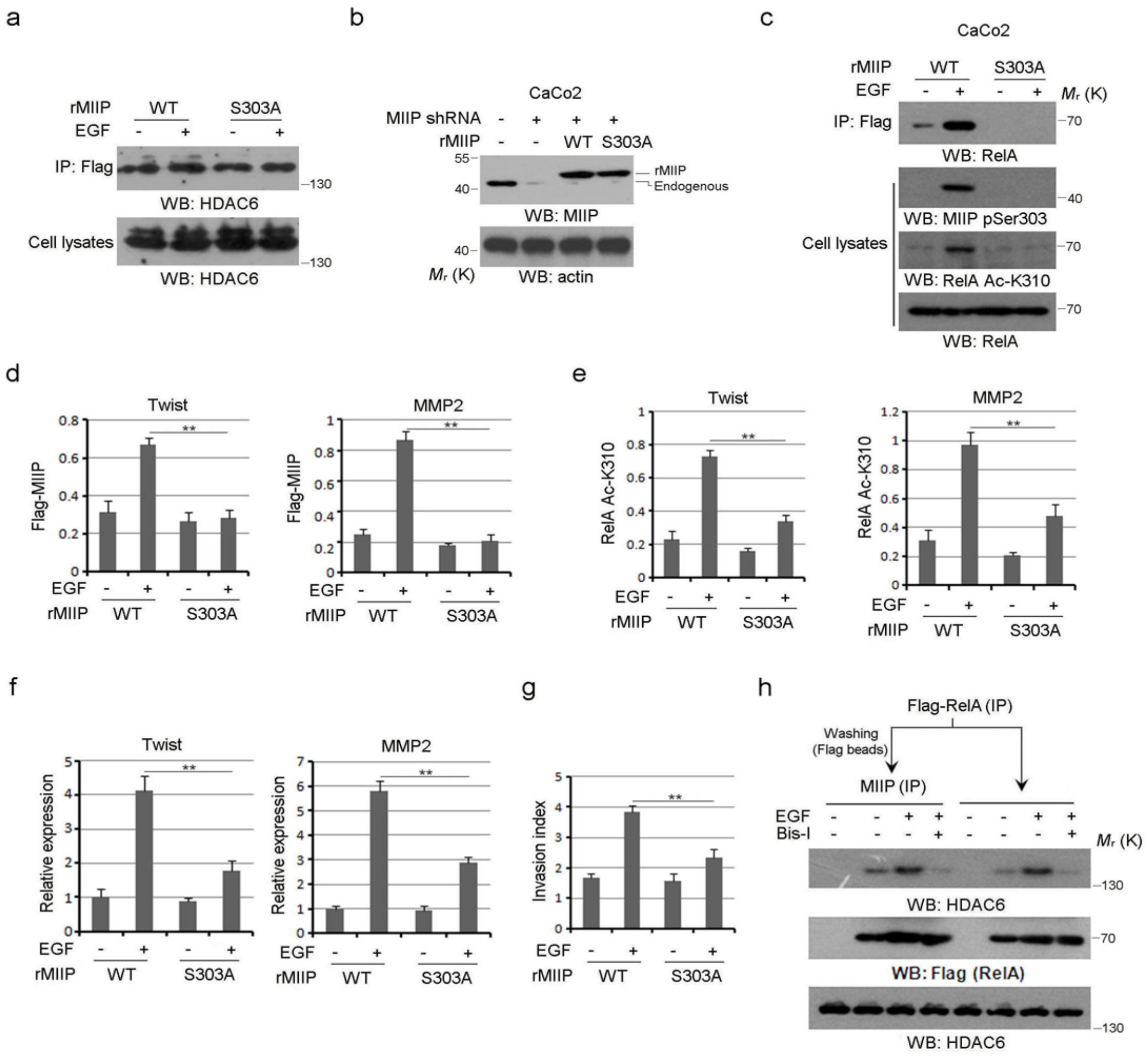

Supplementary Figure 4. MIIP prevent HDAC6-mediated RelA deacetylation.

(**a**) HCT116 cells expressed with WT rMIIP or rMIIP S303A were treated with or without EGF. (**b**) CaCo2 cells were expressed with a vector for control shRNA or MIIP shRNA and reconstituted with expression of WT rMIIP or rMIIP S303A. (**c**) CaCo2 cells reconstituted with expression of WT MIIP or MIIP S303A were treated with or without EGF (100 ng/ml) for 10 h. (**d, e**) CaCo2 cells with depletion of MIIP, and reconstituted expression of WT rMIIP or rMIIP S303A were treated with or without EGF (100 ng/ml) for 10 h. ChIP analyses with indicated antibodies were performed. The primers covering RelA binding site of MMP2 gene promoter region were used for the q-PCR. The Y axis shows the value normalized to the input. (**f**) CaCo2 cells with depletion of MIIP, and reconstituted expression of WT rMIIP or

rMIIP S303A were treated with or without EGF for 10 h. Relative mRNA levels were analyzed by q-PCR. (**g)** CaCo2 cells with depletion of MIIP, and reconstituted expression of WT rMIIP or rMIIP S303A were treated with or without EGF (100 ng/ml). Cell invasion assays were performed. (**h)** CaCo2 cells were pretreated with or without Bis-l for 1 h prior to EGF (100 ng/ml) treatment for 30 min. Cellular extracts subjected to immunoprecipitation with an anti-Flag, followed by Flag-beads washing and a second immunoprecipitation with an anti-MIIP (lane 1-4 from left). Cellular extracts subjected to immunoprecipitation with an anti-Flag (lane 5-8 from left). In a,b, c and h, Immunoprecipitation and immunoblotting analyses were performed using the indicated antibodies. Data represent 1 out of 3 experiments. In d-g, ** represents $P<0.01$ between the indicated groups.

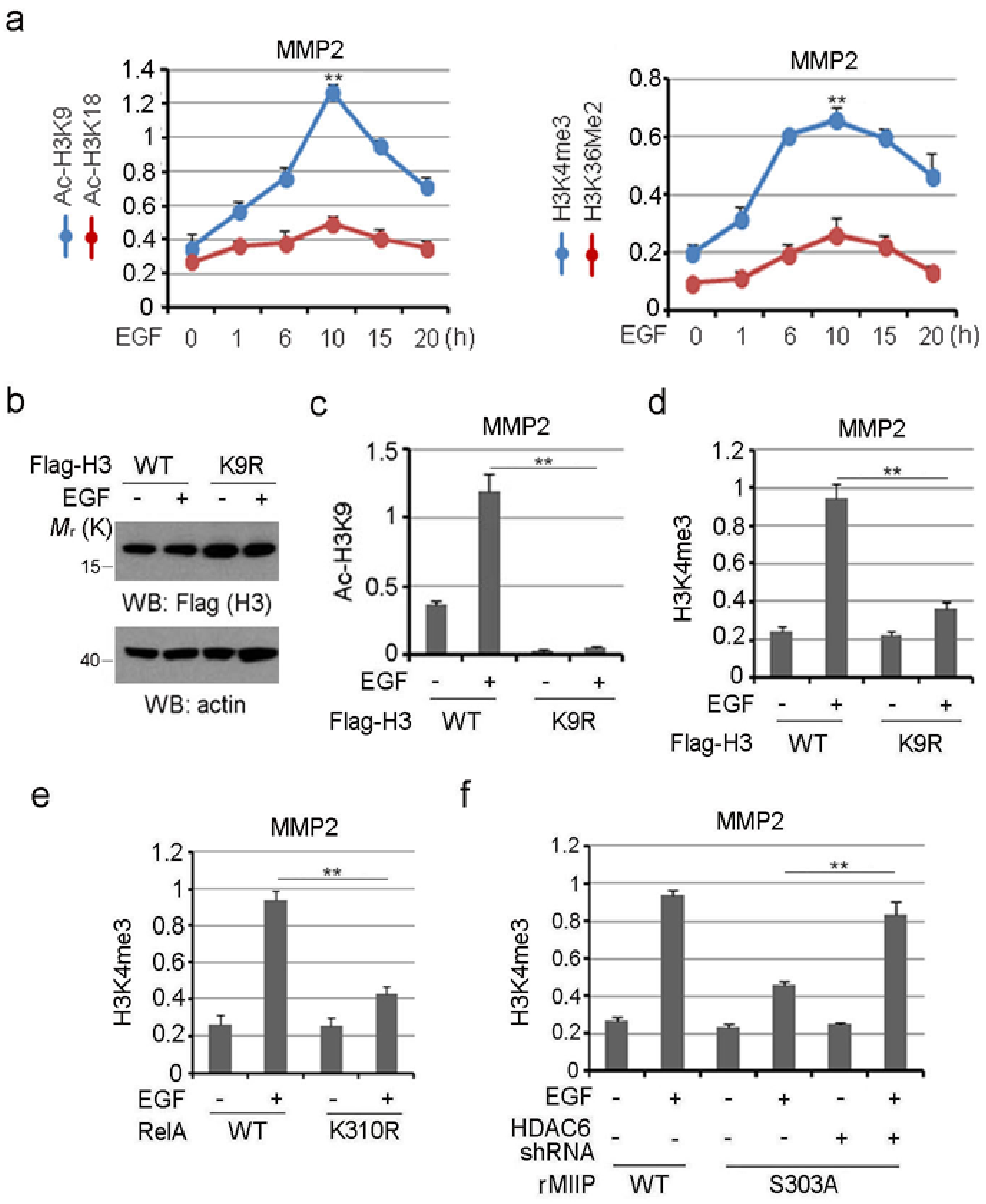

Supplementary Figure 5. MIIP-RelA Interaction Facilitates H3 Lysine9 Acetylation at Promoter Region. **(a)** HCT116 cells were treated with or without EGF for indicated length of time. **(b)** HCT116 cells expressed with WT H3 or H3 K9R were treated with or without EGF. Immunoprecipitation and immunoblotting analyses were performed using the indicated antibodies. Data represent 1 out of 3 experiments. **(c, d)** HCT116 cells expressed with WT H3 or H3 K9R were treated

with or without EGF. **(e)** HCT116 cells expressed with WT RelA or RelA K310R were treated with or without EGF for 10 h (100 ng/ml). **(f)** HCT116 cells expressed with WT MIIP or MIIP S303A were overexpressed with or without HDAC6; cells were treated with or without EGF (100 ng/ml) for 10 h. In a, c-f, ChIP analyses with indicated antibodies were performed. The primers covering RelA binding site of MMP2 gene promoter region were used for the q-PCR. The Y axis shows the value normalized to the input. In a, c-f, the values are presented as mean ± s.e.m. (n=3 independent experiments), *P<0.05 and **P<0.01 between the indicated groups.

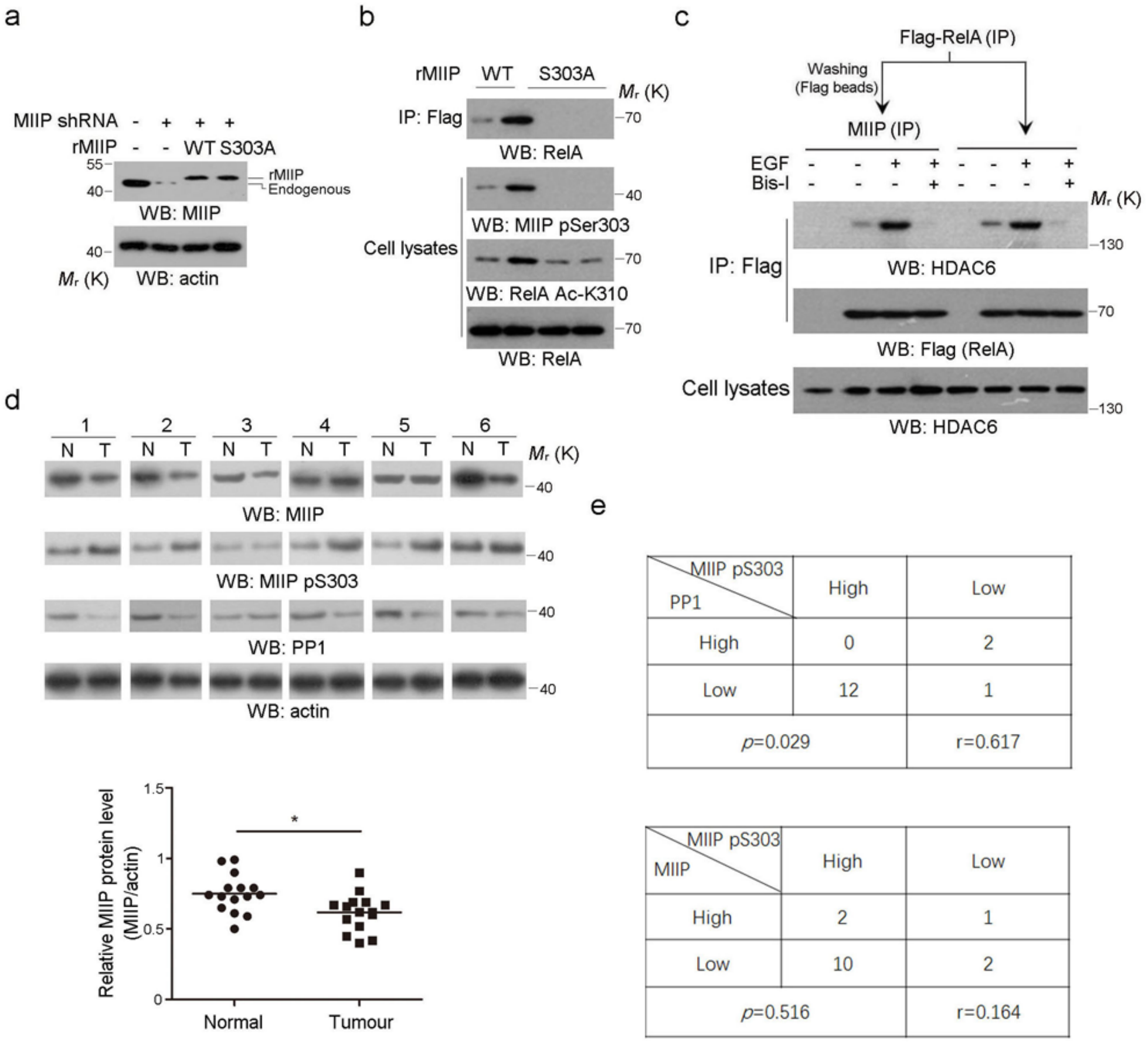

MIIP pS303 / PP1	High	Low
High	0	2
Low	12	1
p=0.029		r=0.617

MIIP pS303 / MIIP	High	Low
High	2	1
Low	10	2
p=0.516		r=0.164

Supplementary Figure 6. MIIP-S303 phosphorylation is required for tumour metastasis and is related to poor prognosis in human colorectal cancer. (a) SW620 cells were expressed with a vector for control shRNA or MIIP shRNA and reconstituted with expression of WT rMIIP or rMIIP S303A. **(b)** SW620 cells reconstituted with expression of WT MIIP or MIIP S303A were treated with or without EGF (100 ng/ml) for 10 h. **(c)** SW620 cells were pretreated with or without Bis-l for 1 h prior to EGF (100 ng/ml) treatment for 30 min. Cellular extracts subjected to immunoprecipitation with an anti-Flag, followed by Flag-beads washing and a second immunoprecipitation with an anti-MIIP (lane 1-4 from left). Cellular extracts subjected to immunoprecipitation with an anti-Flag (lane 5-8 from left). (d) Representative immunoblotting analysis of expression of MIIP, MIIP pS303, and PP1

in tumour tissues (T) versus the adjacent normal tissues (N) from human colorectal cancer specimens (upper panel). Scatter plot analysis of the expression levels of MIIP protein (bottom panel). MIIP expression levels were quantified by densitometry. The expression of MIIP was normalized to actin. Statistical significance was determined by a two-tailed, paired Student t test. * represents $p < 0.05$. (e) Correlation Analysis of MIIP pS303 with MIIP (up) and PP1 (down) expression in 15 colorectal cancers compared with their adjacent normal tissues. High, expression level was high in the colorectal cancer compared with the adjacent normal tissue. Low, expression level was low in the colorectal cancer compared with the adjacent normal tissue. Fisher’s exact test is chosen when the minimum expected count is less than 1 or the total number is less than 40. In a-d, immunoprecipitation and immunoblotting analyses were performed using the indicated antibodies. Data represent 1 out of 3 experiments.

Fig. 1

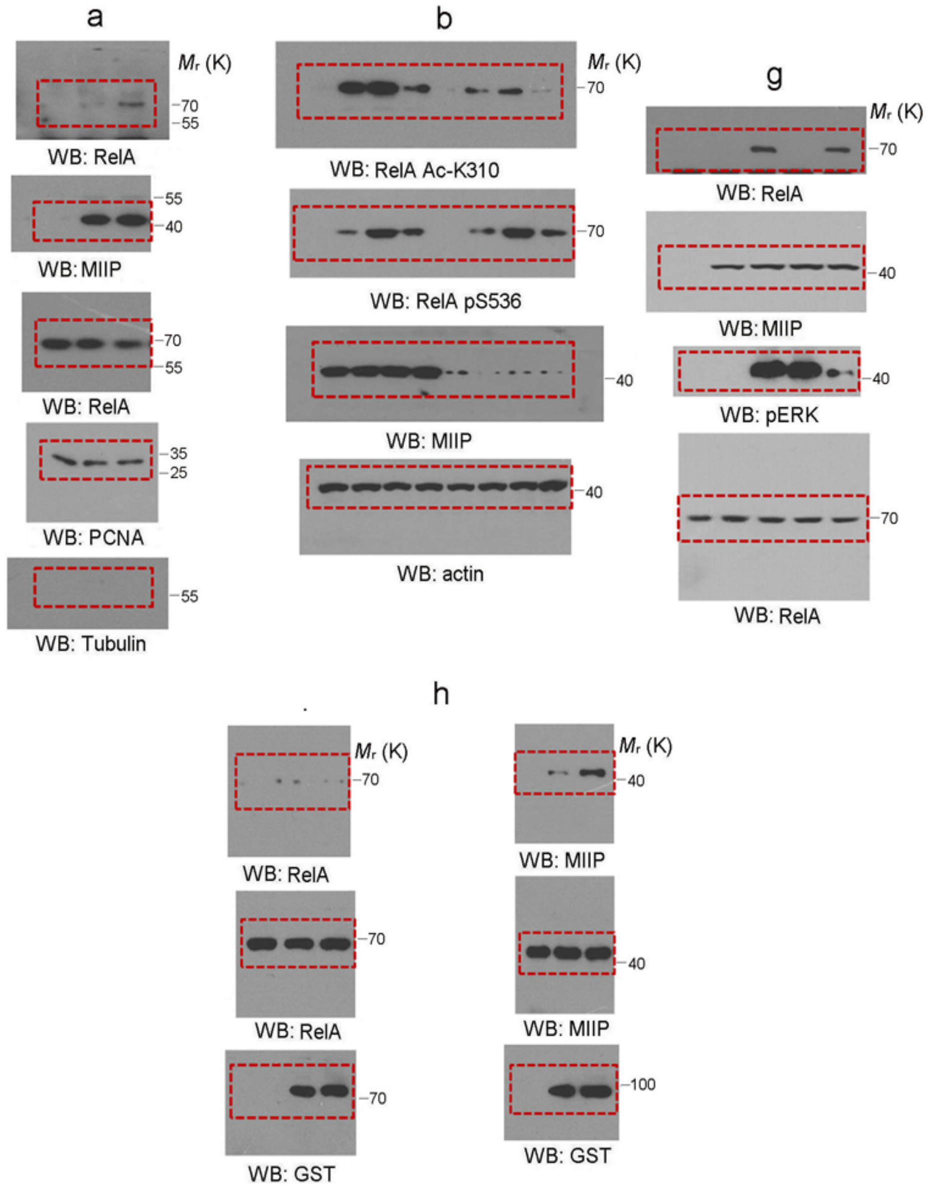
a
b
g
h
Mr (K)
70
55
40
35
25
100
WB: RelA
WB: MIIP
WB: PCNA
WB: Tubulin
WB: RelA Ac-K310
WB: RelA pS536
WB: actin
WB: pERK
WB: GST

Fig. 2

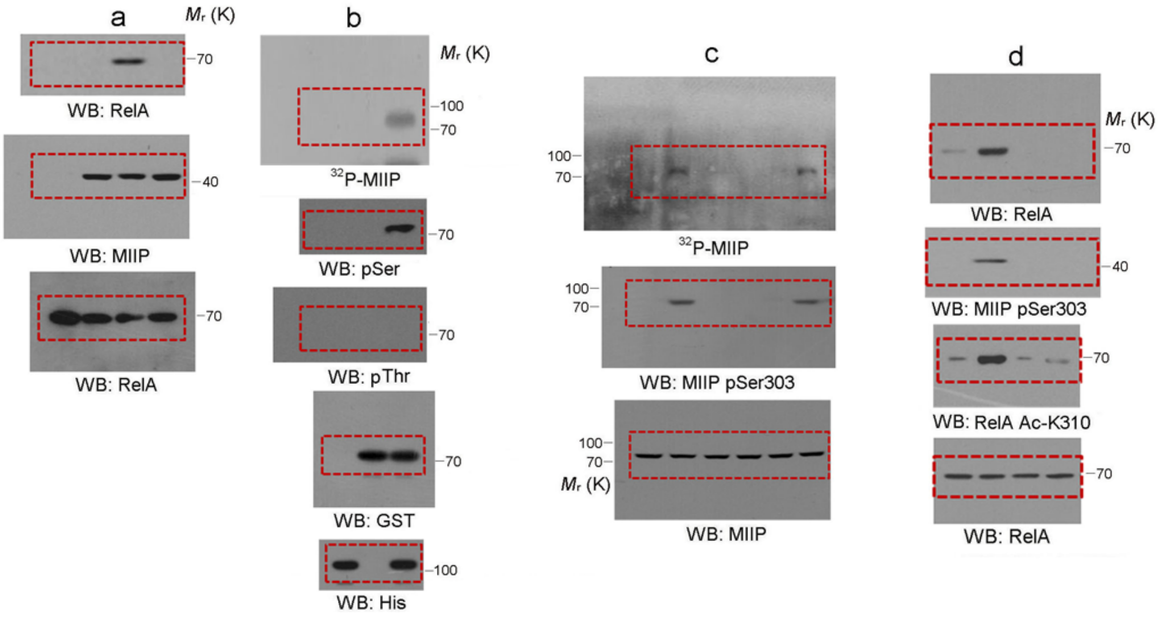

a
b
c
d
M_r (K)
70
40
100
WB: RelA
WB: MIIP
^{32}P-MIIP
WB: pSer
WB: pThr
WB: GST
WB: His
WB: MIIP pSer303
WB: RelA Ac-K310

Fig. 3

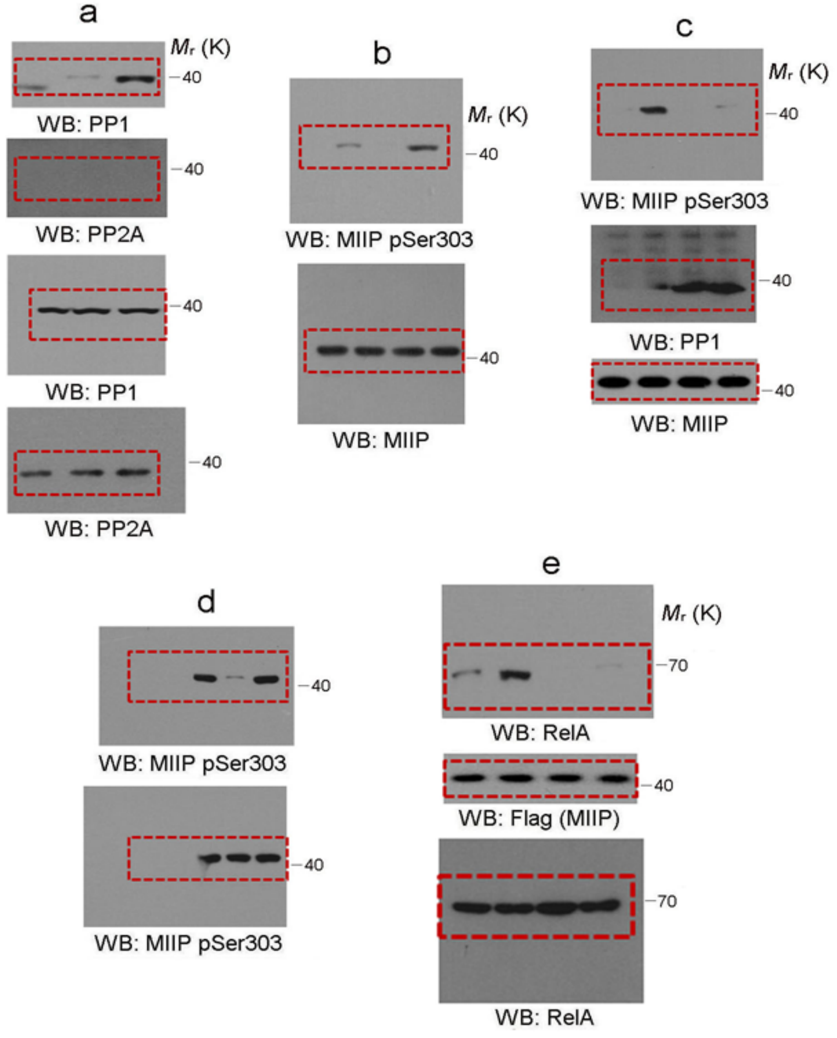
a
Mr (K)
40
WB: PP1
40
WB: PP2A
40
WB: PP1
40
WB: PP2A
b
Mr (K)
40
WB: MIIP pSer303
40
WB: MIIP
c
Mr (K)
40
WB: MIIP pSer303
40
WB: PP1
40
WB: MIIP
d
40
WB: MIIP pSer303
40
WB: MIIP pSer303
e
Mr (K)
70
WB: RelA
40
WB: Flag (MIIP)
70
WB: RelA

Fig. 4

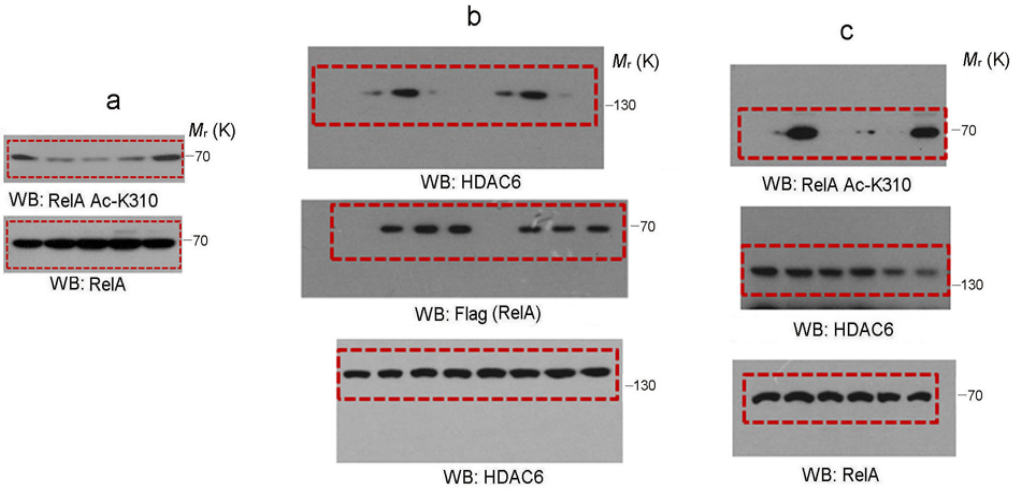

Fig. 5

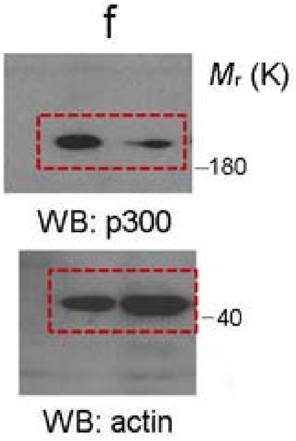

Fig. 6

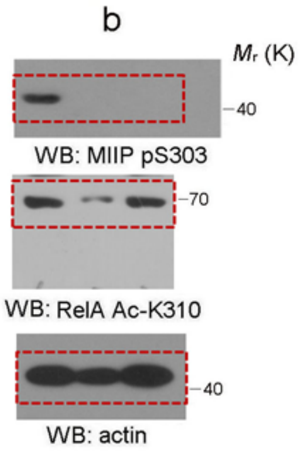

S1

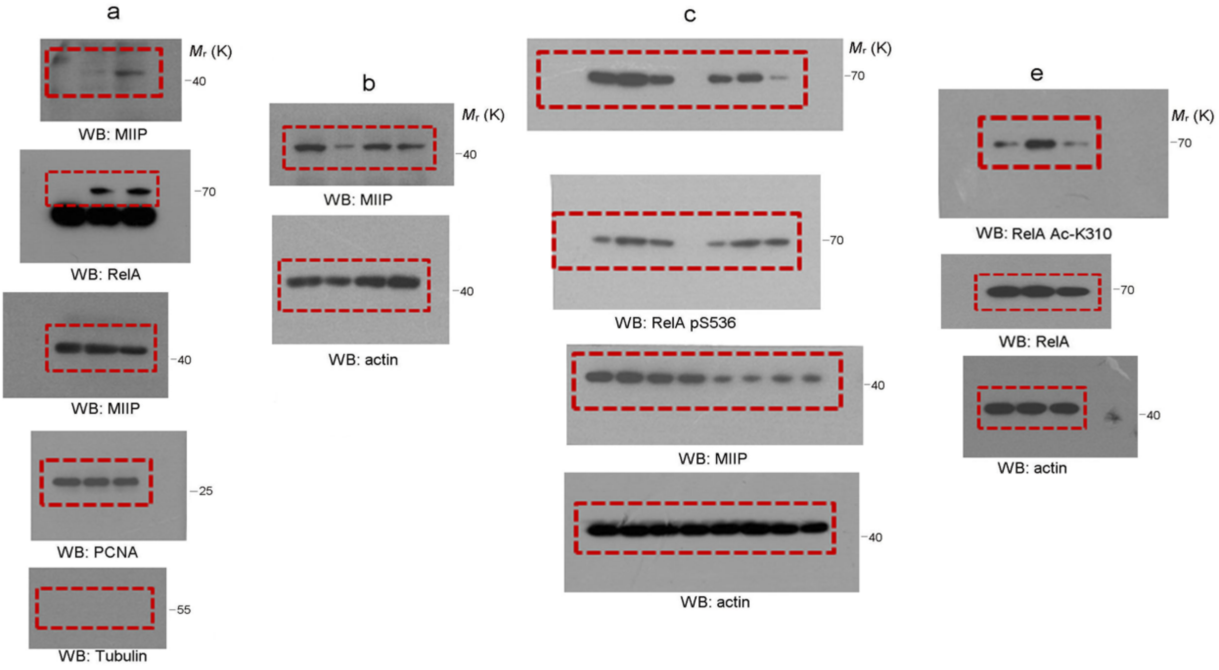

a
M_r (K)
40
WB: MIIP
70
WB: RelA
40
WB: MIIP
25
WB: PCNA
55
WB: Tubulin
b
M_r (K)
40
WB: MIIP
40
WB: actin
c
M_r (K)
70
70
WB: RelA pS536
40
WB: MIIP
40
WB: actin
e
M_r (K)
70
WB: RelA Ac-K310
70
WB: RelA
40
WB: actin

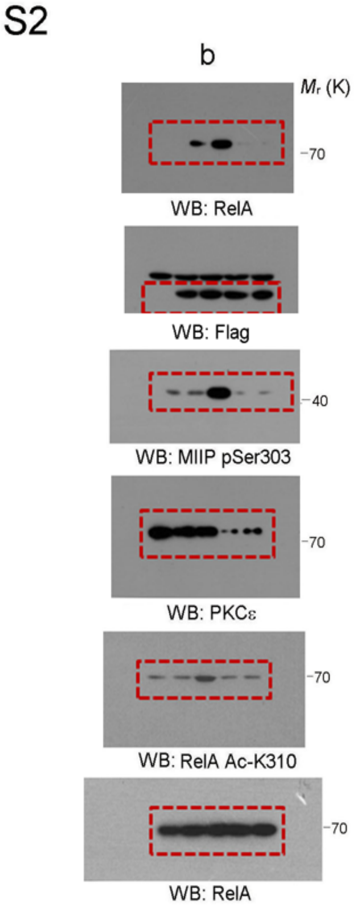
S2
b
M_r (K)
70
WB: RelA
WB: Flag
40
WB: MIIP pSer303
70
WB: PKCε
70
WB: RelA Ac-K310
70
WB: RelA

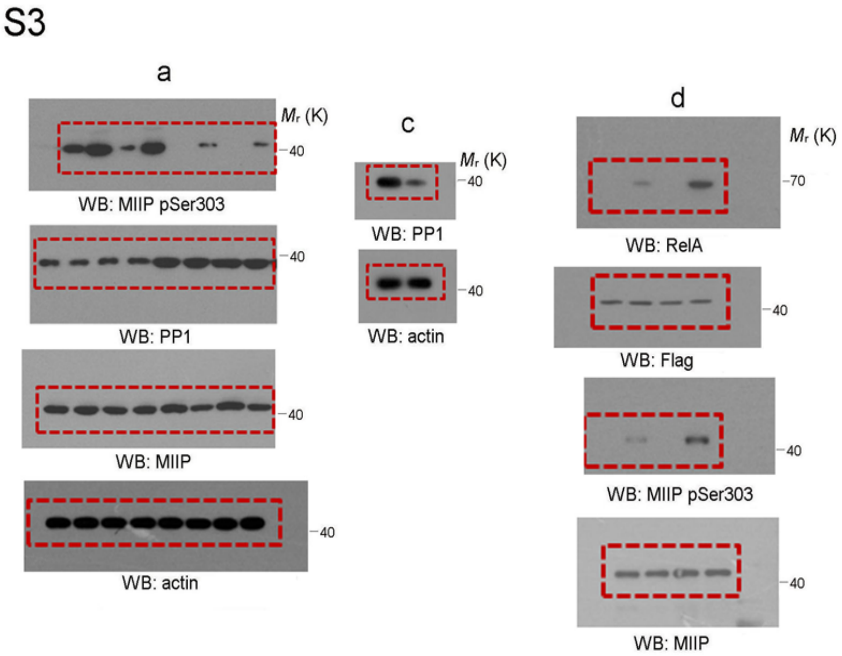
S3
a
M_r (K)
40
WB: MIIP pSer303
40
WB: PP1
40
WB: MIIP
40
WB: actin
c
M_r (K)
40
WB: PP1
40
WB: actin
d
M_r (K)
70
WB: RelA
40
WB: Flag
40
WB: MIIP pSer303
40
WB: MIIP

S4

c

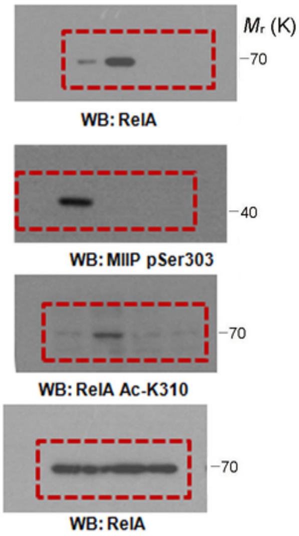

h

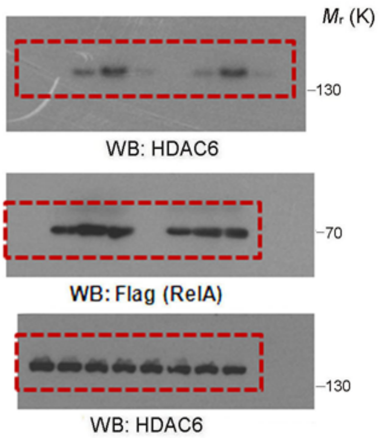

S5

b

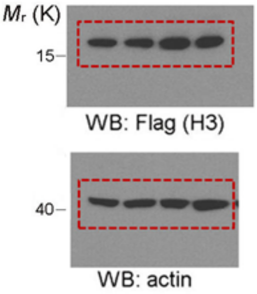

S6

b

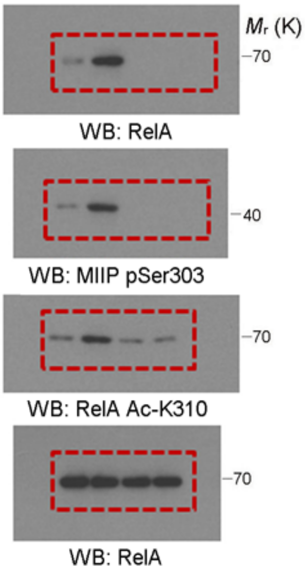

c

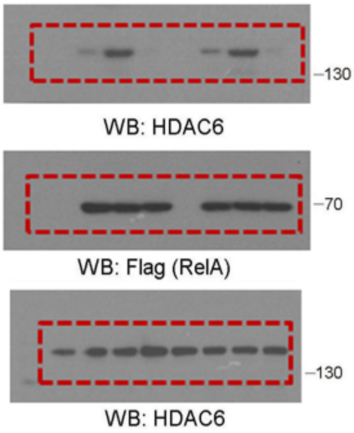

Supplementary Figure 7. Unprocessed original scans of blots.

Supplementary Table 1. Clinicopathological characteristics of 182 patients with colorectal cancer.

Total (n)	182
Gender (n)	
Male	116
Female	66
Age (year), mean±SEM	63±12
Tumor Location (n)	
Right colon	37
Left colon	60
Rectum	85
Differentiation (n)	
Poorly	7
Level II	111
Level II-III	64
T stage (n)	
1	2
2	31
3	148
4	1
N stage (n)	
0	107
1	51
2	24
M stage (n)	
0	162
1	20
MIIP pS303 expression	
High	116
Low	66
